# Two-Electron Reductive Carbonylation of Terminal Uranium(V) and Uranium(VI) Nitrides to Cyanate by Carbon Monoxide**

**DOI:** 10.1002/anie.201406203

**Published:** 2014-07-30

**Authors:** Peter A Cleaves, David M King, Christos E Kefalidis, Laurent Maron, Floriana Tuna, Eric J L McInnes, Jonathan McMaster, William Lewis, Alexander J Blake, Stephen T Liddle

**Affiliations:** School of Chemistry, University of Nottingham, University ParkNottingham, NG7 2RD (UK); LPCNO, CNRS & INSA, Université Paul Sabatier135 Avenue de Rangueil, 31077 Toulouse (France); School of Chemistry and Photon Science Institute, University of ManchesterOxford Road, Manchester, M13 9PL (UK)

**Keywords:** carbon monoxide, carbonylation, cyanates, nitrides, uranium

## Abstract

Two-electron reductive carbonylation of the uranium(VI) nitride [U(Tren^TIPS^)(N)] (**2**, Tren^TIPS^=N(CH_2_CH_2_NSi*i*Pr_3_)_3_) with CO gave the uranium(IV) cyanate [U(Tren^TIPS^)(NCO)] (**3**). KC_8_ reduction of **3** resulted in cyanate dissociation to give [U(Tren^TIPS^)] (**4**) and KNCO, or cyanate retention in [U(Tren^TIPS^)(NCO)][K(B15C5)_2_] (**5**, B15C5=benzo-15-crown-5 ether) with B15C5. Complexes **5** and **4** and KNCO were also prepared from CO and the uranium(V) nitride [{U(Tren^TIPS^)(N)K}_2_] (**6**), with or without B15C5, respectively. Complex **5** can be prepared directly from CO and [U(Tren^TIPS^)(N)][K(B15C5)_2_] (**7**). Notably, **7** reacts with CO much faster than **2**. This unprecedented f-block reactivity was modeled theoretically, revealing nucleophilic attack of the π* orbital of CO by the nitride with activation energy barriers of 24.7 and 11.3 kcal mol^−1^ for uranium(VI) and uranium(V), respectively. A remarkably simple two-step, two-electron cycle for the conversion of azide to nitride to cyanate using **4**, NaN_3_ and CO is presented.

In contrast to a wealth of terminal d-block nitrides,[1] the uranium nitride bond was, until recently,[2] prominent by its absence outside of spectroscopic studies.[3] Molecular uranium nitrides prepared on a large scale exhibited nitrides bridging two to four metal ions, were protected by a covalently bound borane, or decomposed by ligand C=H activation when generated by photolysis.[4] In 2012, as part of our studies of uranium=ligand multiple bonds,[5] we reported the uranium(V) nitride [U(Tren^TIPS^)(N)][Na(12C4)_2_] (**1**, Tren^TIPS^=N(CH_2_CH_2_NSi*i*Pr_3_)_3_), and its oxidation to the uranium(VI) nitride [U(Tren^TIPS^)(N)] (**2**).[6] However, as a consequence of this prior paucity of uranium nitrides, there are no systematic reactivity data so the reactivity trends of uranium nitrides remains unknown. We showed that **1** reacts with Me_3_SiCl to afford the trimethylsilylimido derivative, consistent with a nucleophilic nitride.[6a] In contrast, the reactivity of CN with a diuranium μ-nitride[5f] is reminiscent of electrophilic reactivity. Under photolytic conditions, a transient uranium nitride undergoes C=H activation of a C=H bond of a coligand followed by U=C bond migration to the incipient U=NH moiety.[4i] As CO is ambiphilic, and a key molecule in industry and the environment, we became interested in examining any reactivity that our neutral and anionic terminal uranium nitrides might exhibit toward CO, as the nature of uranium nitrides in varied oxidation states is yet to be established and carbonylation of d-block nitrides is a very rare and recent accomplishment.[7]

Here, we report the first comparative study of the reactivity of a uranium nitride bond and show that the reaction with CO consistently proceeds through nitride nucleophilic attack to give cyanate; the latter is an important, fundamental inorganic functional group with wide-ranging industrial applications.[8] Interestingly, whether the newly formed cyanate remains bound to uranium depends on the uranium oxidation state and the presence, or absence, of a crown ether. The uranium(V)=nitride bond is much more reactive than the uranium(VI)=nitride bond, and the individual reaction steps allow us to construct a remarkably simple two-step, two-electron synthetic cycle for the conversion of azide to nitride to cyanate.

Stirring a toluene solution of neutral **2** under an atmosphere of CO for 16 hours resulted in the isolation of the pale-green uranium(IV) cyanate [U(Tren^TIPS^)(NCO)] (**3**) in 76 % yield after work-up (Scheme 1).[9] Complex **3** exhibits a strong absorption at 2187 cm^−1^ in the FTIR spectrum, which is characteristic of a metal cyanate, and comparable to the corresponding value of 2185 cm^−1^ of [U{tacn(O-Ar^Ad^)_3_}(NCO)].[10] The magnetic moment of **3** in solution at 298 K is 2.50 μ_B_; in the solid state the magnetic moment is 2.41 μ_B_ at 298 K, and decreases to 0.44 μ_B_ at 2.0 K (tending to zero). The electronic absorption spectrum exhibits weak f→f absorptions across the visible and NIR regions (ε≤40 m^−1^ cm^−1^), which together with the magnetic data confirm that carbonylation of **2** to **3** results in a two-electron reduction of uranium(VI) to uranium(IV). For comparative purposes, we found that **3** can be prepared from NaNCO and [U(Tren^TIPS^)(Cl)] in 74 % yield of isolated crystals.[9]

**Scheme 1:**
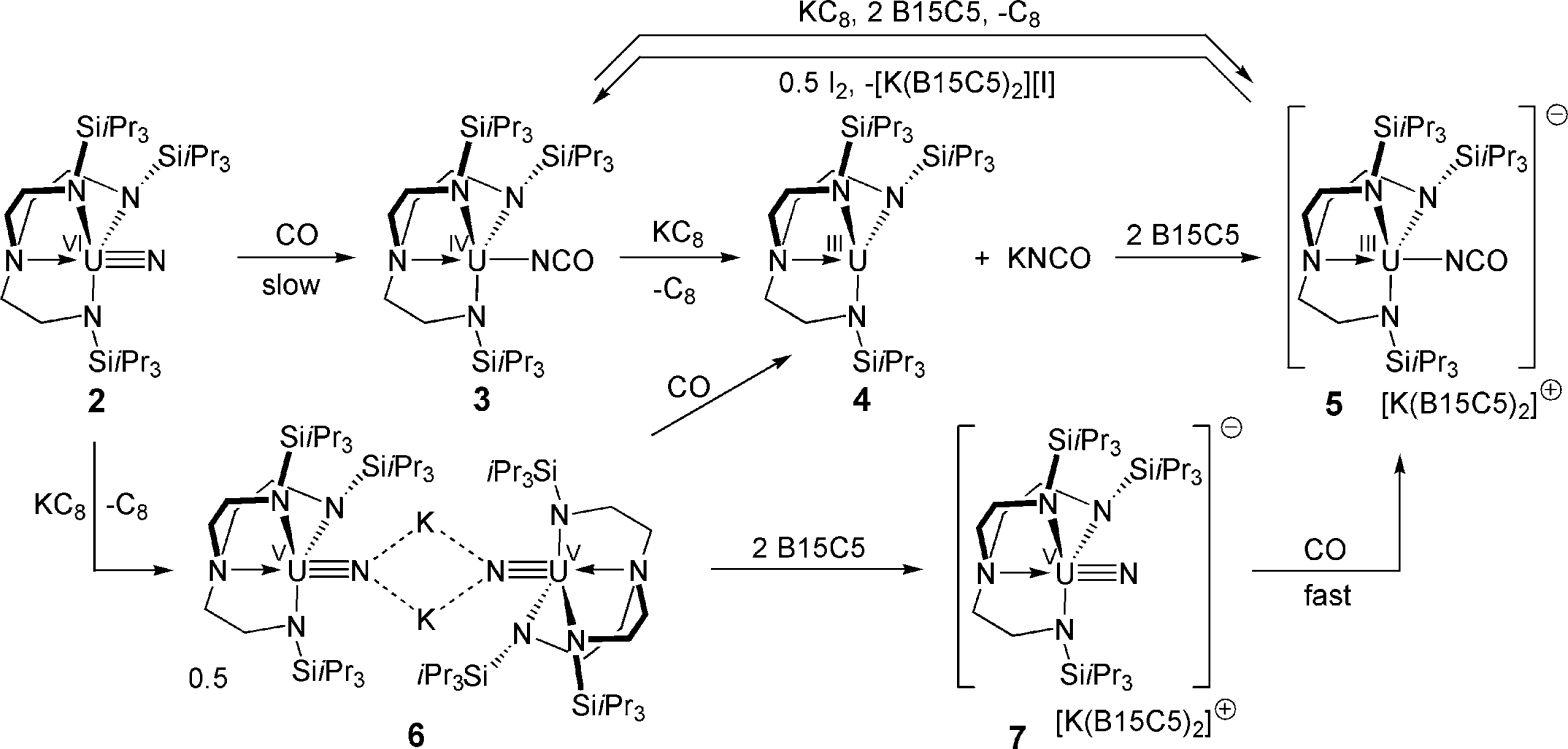
Synthesis of 2–7.

The X-ray crystal structure of **3**[9] (Figure 1 a) shows an essentially linear uranium cyanate unit disordered over two positions (av. U-N-C ∡=173.1°), with a U=N_cyanate_ bond length of 2.338(3) Å (compare with U=N_amide_=2.251 Å (av.); U=N_amine_=2.620(3) Å) which compares well to the U=N_cyanate_ bond in [U{tacn(OAr^Ad^)_3_}(NCO)] (2.389(6) Å).[10] The N-bound cyanate assignment in **3** is confirmed by crystallographic refinement and DFT calculations,[9] which show this isomer to be more stable than the *O*-bound isomer by 15.4 kcal mol^−1^. Reductive carbonylation of **2** to **3** is notable for paralleling d-block terminal nitride reactivity, but this is an exceptionally rare reaction.[7] There are only two other, but different, examples of NCO formation at uranium; [U{η^8^-C_8_H_6_(1,4-Si*i*Pr_3_)_2_}(η^5^-C_5_Me_5_)]_2_(μ-NCO)_2_ was formed by bimetallic reductive activation of NO and CO,[11] and [U{tacn(OAr^Ad^)_3_}(NSiMe_3_)] reacted with CO to give [U{tacn(OAr^Ad^)_3_}(NCO)] with elimination of Me_3_SiSiMe_3_.[10], [12]

**Figure 1:**
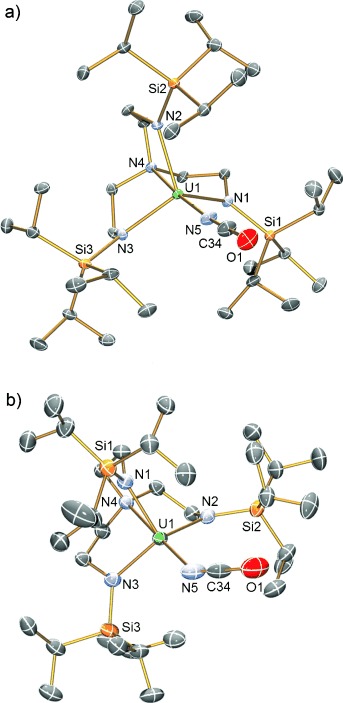
Molecular structures of 3 (a) and the anion component of 5 (b). Displacement ellipsoids set to 40 %; hydrogen atoms and disorder components omitted for clarity.

One-electron reduction of **3** with KC_8_ follows two divergent routes, depending on the reaction conditions (Scheme 1),[9] but notably these reactions do not include reductive decarbonylation to afford **6** (see below), as has been uniquely observed for a niobium cyanate;[13] this can be ascribed to the weaker nature of the U=N bond that would be formed compared to a Nb=N bond. When **3** is reduced with KC_8_ in toluene, the solution immediately turns from brown to dark purple, which is characteristic of uranium(III), with concomitant extrusion of KNCO (υ_NCO_=2130 cm^−1^).[14] In this regard, this denitrification reactivity is similar to that reported for a vanadium nitride.[7a] Filtration of the toluene-soluble [U(Tren^TIPS^)] (**4**), identified by comparison of its characterization data with an authentic sample, from the KNCO precipitate affords both compounds in essentially quantitative yields. Notably, the addition of KNCO to **4** does not result in the reverse reaction. When **3** is reduced by KC_8_ in the presence of two equivalents of benzo-15-crown-5 ether (B15C5), or for an independent synthesis two equivalents of B15C5 are added to a mixture of KNCO and **4**, the solutions turn dark green and the cyanate anion binds to uranium to give the uranium(III)-separated ion pair cyanate [U(Tren^TIPS^)(NCO)][K(B15C5)_2_] (**5**), which can be isolated as dark green crystals in 45 % yield.[9]

The FTIR spectrum of **5** exhibits a broad absorption centered at 2172 cm^−1^, which is consistent with a uranium=cyanate linkage. The electronic absorption spectrum of **5** exhibits moderate absorptions at 573 and 679 nm (ε≈360 m^−1^ cm^−1^) as well as weaker absorptions across the visible and NIR regions (ε≤60 m^−1^ cm^−1^), which are assigned as f→d and f→f transitions of uranium(III), respectively. The solution magnetic moment of **5** at 298 K is 2.76 μ_B_ and the solid-state magnetic moment at 298 K is 2.59 μ_B_, decreasing to 1.13 μ_B_ at 2.0 K; combined with EPR studies,[9] this confirms the uranium(III) formulation that is a magnetic doublet at low temperature.

The X-ray crystal structure of **5** (Figure 1 b)[9] confirms the formulation. The salient feature of **5** is a bent uranium=cyanate linkage with the cyanate CO portion disordered over two positions (av. U-N-C ∡=138.1°) and a U=N_cyanate_ bond length of 2.456(7) Å (U=N_amide_=2.359 Å (av.); U=N_amine_=2.685(5) Å); these bond lengths are approximately 0.1 Å longer than the corresponding distances in **3**, commensurate with the change in uranium oxidation state from (IV) to (III). As for **3**, the cyanate in **5** is found to be N-bound from crystallographic refinement, and this isomer is calculated to be 12.6 kcal mol^−1^ more stable than the O-bound isomer.[9]

As CO effects a two-electron reductive carbonylation of neutral **2** to give **3**, and further one-electron reduction affords **4** and free KNCO, or **5** when B15C5 is present, we surmised that reactions giving uranium(III) products should be directly accessible from anionic uranium(V) nitrides (Scheme 1).[9] Accordingly, we prepared [{U(Tren^TIPS^)(N)K}_2_] (**6**)[9] and found that when a solution of **6** in toluene is stirred under an atmosphere of CO, two-electron reductive carbonylation occurs to give **4** and free KNCO in around 90 % yield. Interestingly, whereas the reaction between **2** and CO is slow and proceeds over several hours, the reaction between **6** and CO is immediate, even at −78 °C. Addition of B15C5 at any stage resulted in isolation of **5**. To independently verify these reactions, we prepared [U(Tren^TIPS^)(N)][K(B15C5)_2_] (**7**) from **6** and two equivalents of B15C5 and observed an immediate reaction of a toluene solution of **7** with CO, even at −78 °C, to give **5** in 94 % yield of isolated crystals; this is an unusual reaction and suggests that the uranium(V) nitride is highly oxidizing. Lastly, treatment of **5** with half a molar equivalent of I_2_ quantitatively regenerates **3**.

In order to probe the origin of the difference in reaction rates of uranium(VI) (**2**) and uranium(V) nitrides (**7**) with CO, and to determine the mechanism(s) by which nitride reductive carbonylation occurs, given the ambiphilic nature of CO, we probed the reaction profiles using DFT calculations (Figure 2). Starting from **2** or **7**, the reactions are kinetically accessible and thermodynamically favorable and can be described as nucleophilic attack of the nitride to the incoming CO molecule, as revealed by the molecular orbitals at the transition state (TS). Indeed, the HOMO (^**VI**^**TS_I-II_**) or the HOMO-1 (^**V**^**TS_7-II_**) exhibit an overlap between the nitride *p*-lone pair and the π* orbital of CO. This is somewhat different from a [2+2] addition reaction, which may be interpreted as CO addition to the nitride, and is in-line with experiments, as **2** and **7** do not undergo electrophilic addition to PMe_3_. An important feature of these reactions is that the spin-state change only occurs after the TS. Indeed, for **2**, a TS in the triplet spin state was located 20 kcal mol^−1^ higher than the one reported here.[9] The experimental difference of reaction rates between **2** and **7** is corroborated theoretically and explained by CO precoordination. For **2**, because of the smaller size of uranium(VI), CO must approach closer to the metal to react than for uranium(V); this coordination is energetically costly (endergonic by 15.3 kcal mol^−1^), thus making the barrier higher for uranium(VI) than for uranium(V). The uranium(VI) center in ^**VI**^**I** is f^0^, whereas there is no such intermediate for f^1^ uranium(V), and instead **7** converts straight to ^**V**^**TS_7-II_** without adduct formation; thus, uranium backbonding to CO appears to play no role in the observed reactivity.

**Figure 2:**
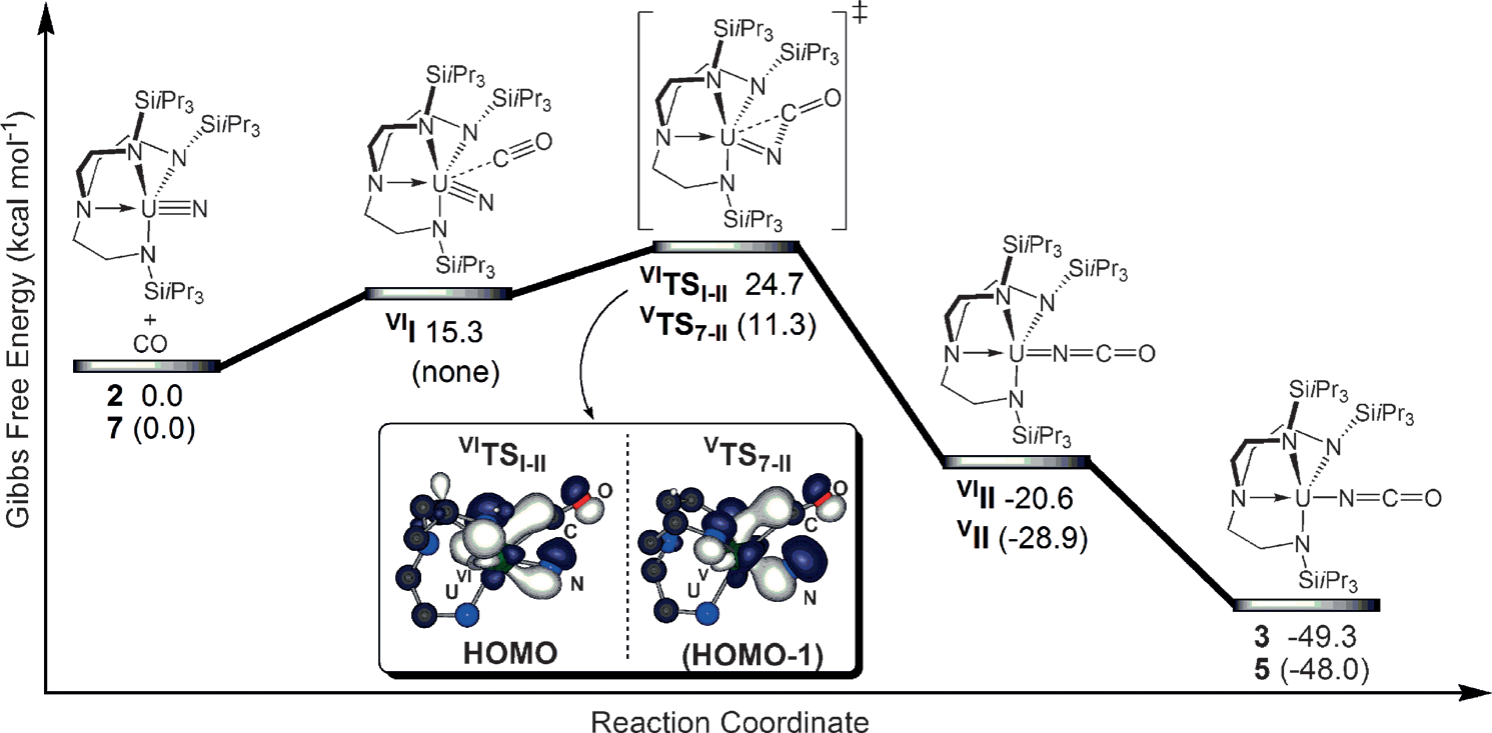
Gibbs free energy reaction profile for the reductive carbonylation of neutral 2. Numbers in parenthesis correspond to the anionic portion of 7. Full models were calculated, peripheral substituents were omitted for clarity in molecular orbital plots.

The reactions described above suggested that a synthetic cycle for nitride to cyanate conversion should be possible based on a two-electron U^III^-U^V^ redox couple (Schee 2). The two-electron reductive carbonylation/denitrification of **6** to **4** and KNCO generates a uranium(III) complex that is known to undergo a two-electron oxidation with azide, specifically NaN_3_, to generate [{U(Tren^TIPS^)(N)Na}_2_] (**8**, compare with **6**). Importantly, **4** does not react with excess CO, unlike [U(Tren^DMBS^)] (Tren^DMBS^=N(CH_2_CH_2_NSiMe_2_-*t*Bu)_3_) which reductively homologates CO to ethyne diolate.[16] As proof-of-concept, we mixed **4** with NaN_3_, in pyridine rather than toluene to overcome the sluggish azide reactivity with **4**, under a CO atmosphere, which resulted in the formation of N_2_ and NaNCO (υ_NCO_=2228 cm^−1^)[7a] and **4**. Encouraged by this result, we repeated this reaction with ten equivalents of NaN_3_, but only one turnover occurred.[9]

**Scheme 2:**
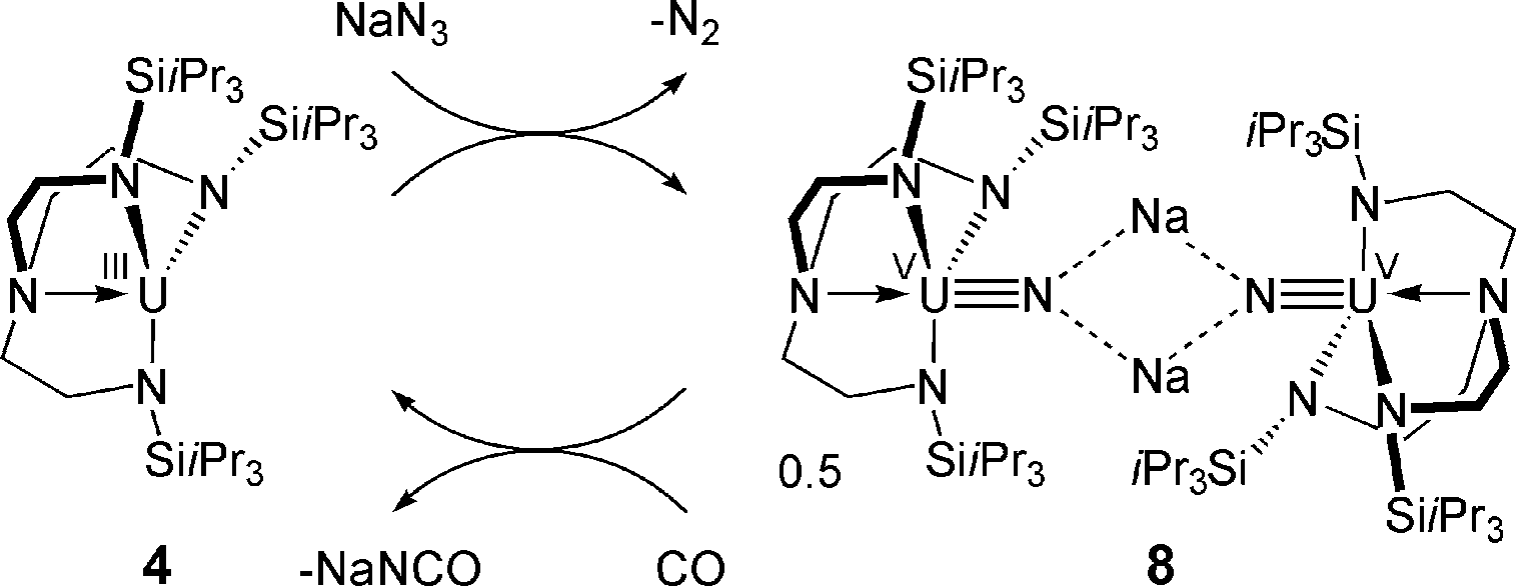
A two-step synthetic cycle for azide to nitride to cyanate conversion.

Although the two-step cycle in Scheme 2 is currently stoichiometric, it is the simplest such cycle reported to date and we believe the limitation is the requirement to use pyridine as solvent to produce the nitride, as **8** is poorly soluble in this solvent. Nevertheless, the reductive carbonylation of a metal nitride remains a very rare transformation,[7a–c] and newly formed substrates usually remain bound to the metal;[7b] only three examples of denitrification to give complete N-atom transfer to a substrate are known to occur and they involve d- or p-block compounds.[7a, 17] Thus, the reactivity in Scheme 2 represents a new precedent in f-block chemistry and highlights surprising similarities of uranium nitride reactivity to d- and even p-block analogues. To make the synthetic cycle catalytic will require careful optimization of the reaction medium, and studies of this are underway.
