# Supplementary material for: Two-Electron Reductive Carbonylation of Terminal Uranium(V) and Uranium(VI) Nitrides to Cyanate by Carbon Monoxide
Source: Angew Chem Int Ed Engl. 2014 Jul 30;53(39):10412–5. doi: 10.1002/anie.201406203 (PMC4497608; doi:10.1002/anie.201406203)
Supplement: Supplementary file 1 [file anie0053-10412-sd1.pdf]

Angewandte Chemie
Eine Zeitschrift der Gesellschaft Deutscher Chemiker

Supporting Information

© Wiley-VCH 2014

69451 Weinheim, Germany

Two-Electron Reductive Carbonylation of Terminal Uranium(V) and Uranium(VI) Nitrides to Cyanate by Carbon Monoxide**

Peter A. Cleaves, David M. King, Christos E. Kefalidis, Laurent Maron, Floriana Tuna, Eric J. L. McInnes, Jonathan McMaster, William Lewis, Alexander J. Blake, and Stephen T. Liddle**

anie_201406203_sm_miscellaneous_information.pdf

- Supporting Information –

Table of Contents

Experimental……………………………………………………………..……………………S2

UV/Vis/NIR Electronic Absorption spectra………………………………………...…………S8

Variable Temperature SQUID Data…………………………………………………………...S8

EPR Data………………………………………………………………………………………..S10

X-Ray Crystallography……………….……………………………………………..……….S11

Density Functional Theory Calculations………………………………………………………S12

References…………………………………………………………………………………S45

Experimental

General

All manipulations were carried out using Schlenk techniques, or an MBraun UniLab glovebox, under an atmosphere of dry nitrogen. Solvents were dried by passage through activated alumina towers and degassed before use or were distilled from calcium hydride. All solvents were stored over potassium mirrors except ethers which were stored over activated 4 A sieves. Deuterated solvent was distilled from potassium, degassed by three freeze-pump-thaw cycles and stored under nitrogen. [U(Tren^{TIPS})(N)] [**2**, Tren^{TIPS} = {N(CH$_2$CH$_2$NSiPr$^i_3$)$_3$}$^{3-}$; Pr^i = CH(CH$_3$)$_2$], [U(Tren^{TIPS})] (**4**), and KC_8 were prepared as described previously.[1-3] Benzo-15-crown-5 ether, KOBu^t and NaN_3 were dried under vacuum for four hours prior to use. All other reagents were used as received.

^{1}H and ^{29}Si NMR spectra were recorded on a Bruker 400 spectrometer operating at 400.2, and 79.5 MHz respectively; chemical shifts are quoted in ppm and are relative to TMS (^{1}H, ^{29}Si). FTIR

spectra were recorded on a Bruker Tensor 27 spectrometer. UV/Vis/NIR spectra were recorded on a Perkin Elmer Lambda 750 spectrometer. Data were collected in 1mm path length cuvettes loaded in an MBraun UniLab glovebox and were run versus the appropriate reference solvent. Static variable-temperature magnetic moment data were recorded in an applied dc field of 0.1 T on a Quantum Design MPMS XL7 superconducting quantum interference device (SQUID) magnetometer using doubly recrystallized powdered samples. Samples were carefully checked for purity and data reproducibility between several independently prepared batches for each compound examined. Care was taken to ensure complete thermalization of the sample before each data point was measured and samples were immobilized in an eicosane matrix to prevent sample reorientation during measurements. Diamagnetic corrections were applied for **3**, **5**, **6** and **7** using tabulated Pascal constants, and data were also corrected for the effect of the blank sample holders (flame sealed Wilmad NMR tube and straw) and eicosane matrix. Solution magnetic moments were recorded at room temperature using the Evans method. Variable temperature (300-5 K) EPR spectra were measured at X-band (ca. 9.5 GHz) on a Bruker EMX spectrometer. Polycrystalline samples were sealed under vacuum in 1 mm i.d. silica tubing, and double-contained for EPR by insertion into an X-band silica tube or PTFE sleeve. Measurements were made on several independently prepared batches, which were also analyzed by SQUID and CHN microanalyses, to ensure reproducibility. Spectra were background corrected against blank sample holders measured under identical conditions. CHN microanalyses were carried out by Tong Liu at the University of Nottingham.

Synthesis of [U(Tren^{TIPS})(NCO)] (3)

Method A: A solution of [U(Tren^{TIPS})(N)] (**2**) (0.50 g, 0.56 mmol) in toluene (10 ml) was degassed, cooled to –78 °C and exposed to an atmosphere of CO. The solution was allowed to warm to room temperature with stirring over 16 hours during which time it slowly turned from red to light brown. The solution was filtered and volatiles were removed *in vacuo* to yield **3** as a brown oily solid. Recrystallization from hot hexanes yielded green crystals of **3**. Yield: 0.38 g, 76%.

Method B: THF (30 ml) was added dropwise to a cold (–78 °C) stirring mixture of [U(Tren^{TIPS})(Cl)] (1.55 g, 1.75 mmol) and NaNCO (0.14 g, 2.15 mmol). The mixture was allowed to warm to room temperature with stirring over 16 hours. The mixture was heated to ~60 °C for 5 mins, and allowed to cool slowly to room temperature. Volatiles were removed *in vacuo,* and the product was extracted into hot hexanes (20 ml) to remove the NaCl precipitate. Volatiles were removed *in vacuo* to afford **3** as a brown solid. Recrystallization from hot hexanes yielded green crystals of **3**. Yield: 1.16 g, 74%.

Method C: A solution of iodine (0.05 g, 0.20 mmol) in toluene (10 ml) was added dropwise to a cold (–78 °C) stirring solution of [U(Tren^{TIPS})(NCO)][K(B15C5)$_2$] (**5**) (0.56 g, 0.38 mmol) in toluene (10 ml). The mixture was allowed to warm to room temperature with stirring over 5 hours. The mixture was filtered to remove the [K(B15C5)$_2$][I] precipitate. Volatiles were removed *in vacuo* to yield **3** as a brown oily solid. Recrystallization from hot hexanes yielded green crystals of **3**. Yield: 0.25 g, 73%. Anal. Calcd for $C_{34}H_{75}N_5OSi_3U$: C 45.77; H 8.47; N 7.85%. Found: C 45.48; H 8.53; N 7.46%. ^{1}H NMR (C_6D_6, 298 K): δ 7.35 (60H, br, C*H*$_2$ + CH(C*H*$_3$)$_2$), 6.51 (s, 9H, C*H*(CH$_3$)$_2$), –30.13 ppm (s, 6H, C*H*$_2$). ^{29}Si{^{1}H} NMR (C_6D_6, 298 K): δ −4.65. FTIR *ν*/cm^{-1} (Nujol): 2361 (w), 2187 (s), 2125 (m), 1343 (m), 1134 (m), 1060 (m), 1052 (m), 990 (m), 736 (s), 675 (m), 633 (m), 552 (m), 517 (m). Magnetic moment (Evans method, C_6D_6, 298 K): μ_{eff} = 2.50 μ_B.

Synthesis of [U(Tren^{TIPS})] (4) and KNCO

Method A: Toluene (30 ml) was added dropwise to a cold (–78 °C) stirring mixture of **3** (1.11 g, 1.24 mmol) and KC_8 (0.17 g, 1.25 mmol). The mixture was allowed to warm to room temperature with stirring over 16 hours. The suspension was allowed to settle for 1 hour, and filtered to afford a dark purple filtrate. The filtrate was washed thoroughly with toluene (3 x 5 ml) until the washings were colorless. Volatiles were removed from the combined washings *in vacuo* to afford **4** as a dark blue-purple solid. Yield of **4** and KNCO: 0.98 and 0.10 g, 93 and 100%, respectively.

Method B: C_6D_6 (0.5 ml) was added to a Young's Tap NMR tube charged with [{U(Tren^{TIPS})(N)K}$_2$] (**6**, see below) (15.3 mg, 8.5 µmol). The solution was freeze-thaw degassed, then exposed to an atmosphere of CO at –78 °C. As the suspension thawed, a dark blue-purple solution formed. ^{1}H NMR studies indicated 90% conversion to **4**.

Control experiment: C_6D_6 (0.5 ml) was added to a Young's Tap NMR Tube charged with **4** (21.0 mg, 24.7 µmol) and KNCO (3.0 mg, 37.0 µmol). The sample was heated at 60 °C for 16 hours. ^{1}H NMR studies and no observable colour change indicated no reaction had taken place.

The identity of **4** was confirmed by comparison to previously reported methods.[1]

Synthesis of [U(Tren^{TIPS})(NCO)][K(B15C5)$_2$] (5)

Method A: C_6D_6 was added to a mixture of **3** (19 mg, 21 µmol) and KC_8 (4 mg, 30 µmol). The mixture was heated to 50 °C for 1 min to afford a dark blue-purple suspension. Benzo-15-crown-5 (13 mg, 48 µmol) was added and an immediate colour change to dark green was observed. The mixture was filtered and volatiles removed *in vacuo* to afford **5** as a dark green oily solid. Yield: 1.58 g, 93%

Method B: Toluene (20 ml) was added to a stirring mixture of benzo-15-crown-5 (1.60 g, 5.95 mmol) and KNCO (0.241 g, 2.97 mmol). The mixture was added dropwise to a cold (–78 °C) stirring solution of **4** (2.53 g, 2.97 mmol) in toluene (20 ml). The mixture was allowed to warm to room temperature with stirring over 16 hours. The solution was filtered and volatiles were removed *in vacuo* to afford **5** as a dark green oil. Storage of this oil at room temperature for 72 hours yielded a small crop of dark green crystals of **5**. Alternatively, suspension of the oil in hexanes (10 ml), followed by storage at –80 °C for 3 hours yielded **5** as a dark green solid. Yield 1.95 g, 45%.

Method C: A solution of [U(Tren^{TIPS})(N)][K(B15C5)$_2$] (**7**, see below) (0.79 g, 0.55 mmol) in toluene (10 ml) was degassed, cooled to –78 °C and exposed to an atmosphere of CO. The solution immediately turned dark green and was allowed to warm slowly to room temperature with stirring over 16 hours. Volatiles were removed *in vacuo* to yield a dark green oil. Hexanes (10 ml) were added and the suspension stored at –80 °C for 3 hours to yield **5** as a dark green solid. Yield: 0.76 g, 94%. Anal. Calcd for $C_{62}H_{115}KN_5O_{11}Si_3U$: C 50.73; H 7.90; N 4.77%. Found: C 50.62; H 7.96; N 4.20%. ^{1}H NMR (C_6D_6, 298 K): δ 8.85 (4H, s, C*H*$_2$), 6.95 (4H, s, crown-C*H*), 6.70 (4H, s, crown-C*H*), 3.45-4.05 (36H, m, crown-C*H*$_2$ + C*H*$_2$), 3.07 (3H, br, C*H*(CH$_3$)$_2$), 2.99 (18H, s, CH(C*H*$_3$)$_2$), 2.32 (6H, s, C*H*(CH$_3$)$_2$), 2.11 (4H, s, C*H*$_2$), 0.73 (36H, s, CH(C*H*$_3$)$_2$). ^{29}Si{^{1}H} NMR (C_6D_6, 298 K): δ –16.45. FTIR ν/cm^{-1} (Nujol): 2195 (s), 2172 (s), 1639 (m), 1504 (s) 1220 (s), 1124 (s), 936 (m), 881 (m), 853 (m), 740 (s). Magnetic moment (Evans method, C_6D_6, 298 K): μ_{eff} = 2.76 μ_B.

Synthesis of [{U(Tren^{TIPS})(N)K}$_2$] (6)

Method A: A solution of **2** (0.12 g, 0.14 mmol) in toluene (15 ml) was added dropwise to KC_8 (0.02 g, 0.15 mmol) at –78 °C with stirring. The mixture was allowed to warm to room temperature with stirring over 16 hours. The mixture was allowed to settle (1 hour) and carefully filtered to afford a dark brown solid that was washed with toluene (3 × 5 ml) until the washings obtained were colourless, at which point these were discarded. The solid was then extracted into hot benzene and quickly filtered through a frit to remove the graphite precipitate. The filtrate was stored at 7 °C for 16 hours to yield dark red crystals of **6** which were isolated by filtration and dried *in vacuo.* Yield: 0.20 g, 16%.

Method B: Benzene (5 ml) was added to a stirring mixture of [{U(Tren^{TIPS})(N)Na}$_2$] (0.32 g, 0.18 mmol) and KO^{t}Bu (0.04 g, 0.36 mmol). The mixture was heated to 80 °C for 2 hours and allowed to cool affording a red precipitate. The precipitate was isolated by filtration, extracted into boiling benzene and filtered through a frit. The filtrate was stored at 7 °C yield **6** as red/orange crystals.

Crystalline yield: 0.10 g, 31%. Anal. calcd for $C_{66}H_{150}N_{10}K_2Si_6U_2$: C 43.88; H 8.37; N 7.75. Found: C 44.18; H 8.38; N 7.62. FTIR ν/cm^{-1} (Nujol): 1344 (w), 1134 (w), 1069 (s), 1056 (s), 992 (w), 932 (s), 880 (m), 851 (w), 780 (m), 746 (s), 671 (m), 626 (w), 571 (w), 545 (w), 515 (w). ^{1}H, ^{29}Si NMR and solution magnetic moment (Evans method) could not be obtained due to the insolubility of **6** in aromatic solvent once isolated.

Synthesis of [U(Tren^{TIPS})(N)][K(B15C5)$_2$] (7)

Toluene (25 ml) was added dropwise to a cold (–78 °C) stirring mixture of **6** (1.63 g, 0.90 mmol) and benzo-15-crown-5 (0.97 g, 3.60 mmol). The red/brown mixture was allowed to warm to room temperature with stirring over 16 hours. The mixture was heated to ~80 °C for 5 mins, and allowed to cool slowly to room temperature and then filtered. The solvent was removed *in vacuo* to yield a brown solid. The product was washed with hexanes (2 x 10 ml) and dried *vacuo* to yield **7** as a brown powder. Red crystals of **7** were isolated from a concentrated solution of **7** in toluene. Yield: 1.91 g, 72%. Anal. calcd for $C_{61}H_{115}KN_5O_{10}Si_3U$: C 50.88; H 8.05; N 4.86;. Found: C 50.93; H 7.85; N 4.49. ^{1}H NMR (C_6D_6, 298 K): δ 38.58 (s, 6H, CH_2), 16.98 (s, 6H, CH_2), 10.03-6.80 (br m, 20H, CH_2, OCH_2, Ar-H), 3.85 (s, 20H, OCH_2), −5.61 (s, 9H, CH(CH$_3$)$_2$), −6.30 (s, 54H, CH(CH_3)$_2$). ^{29}Si{^{1}H} NMR (C_6D_6, 298 K): δ −16.10. FTIR ν/cm^{-1} (Nujol): 1596 (m), 1348 (w), 1333 (w), 1296 (m), 1215 (m), 1127 (s), 1081 (s), 1048 (m), 935 (m), 882 (m), 869 (m), 854 (w), 739 (s), 670 (m), 627 (m), 542 (w), 507 (w). μ_{eff} (Evans method, C_6D_6, 298 K): 1.93 μ_B.

Attempted Synthetic cycle towards the conversion of NaN_3 to NaNCO

Pyridine (10 ml) was added to a cold (–40 °C) stirring mixture of **4** (0.510 g, 0.60 mmol) and NaN_3 (0.388 g, 6.00 mmol). The suspension was freeze-thaw degassed and returned to –40 °C and exposed to an atmosphere of CO. The solution was warmed slowly to room temperature with

stirring for 16 hours, to yield a red solution. The ^{1}H NMR spectrum of the mixture revealed resonances assigned to [U(Tren^{TIPS})(N_3)] and **3**, with the absence of **4**. The FTIR spectrum (Nujol) exhibited absorbances assigned to NaNCO (2228 cm^{-1}), **3** (2187 cm^{-1}), NaN_3 (2121 cm^{-1}) and [U(Tren^{TIPS})(N_3)] (2087 cm^{-1}).

Complexes **3**, **5**, **6**, and **7** were characterized additionally by solid state and variable temperature magnetometry in order to confirm the oxidation states of the uranium ions (see below). The data for the uranium(V)-nitride potassium salts **6** and **7** are similar to those reported for their sodium analogues.[1]

UV/Vis/NIR Electronic Spectra

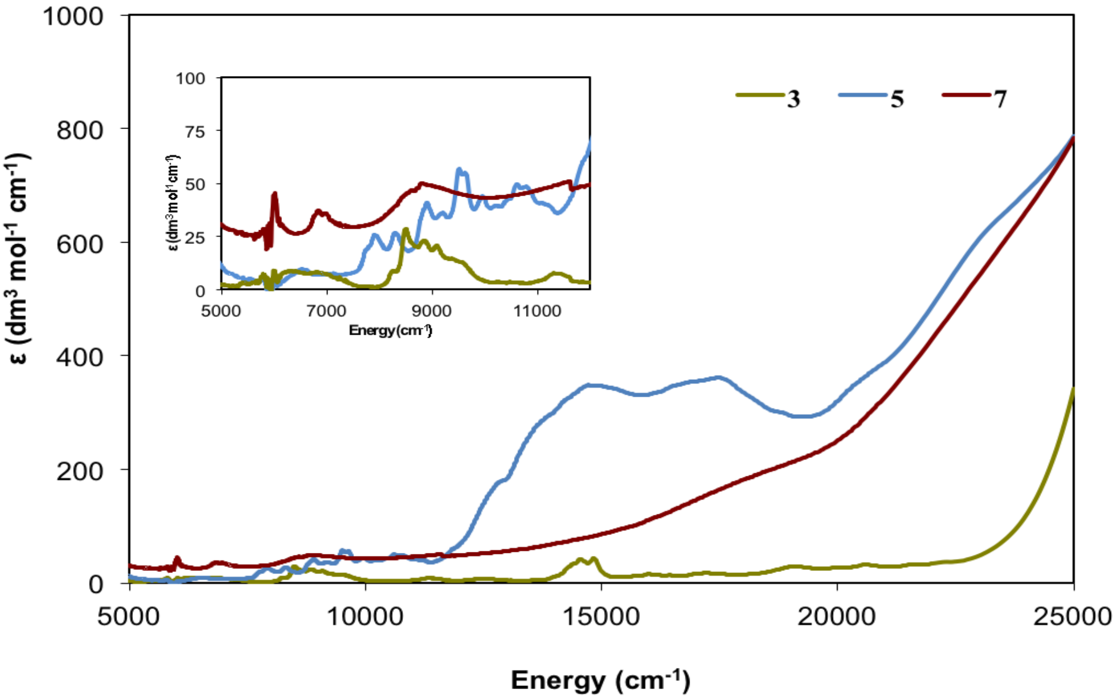

Figure S1. Electronic absorption spectrum of 3, 5 and 7 with zoom in of NIR region.

Variable Temperature SQUID Data

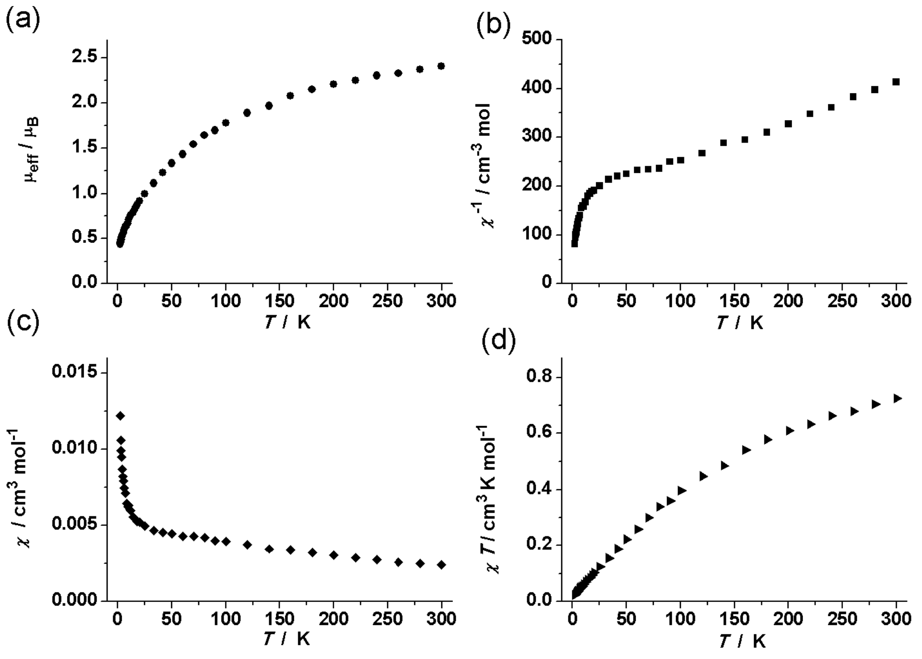

Figure S2. (a) μ_{eff} vs T, (b) 1/χ vs T, (c) χ vs T, and (d) χT vs T of [{U(Tren^{TIPS})(NCO)] (3).

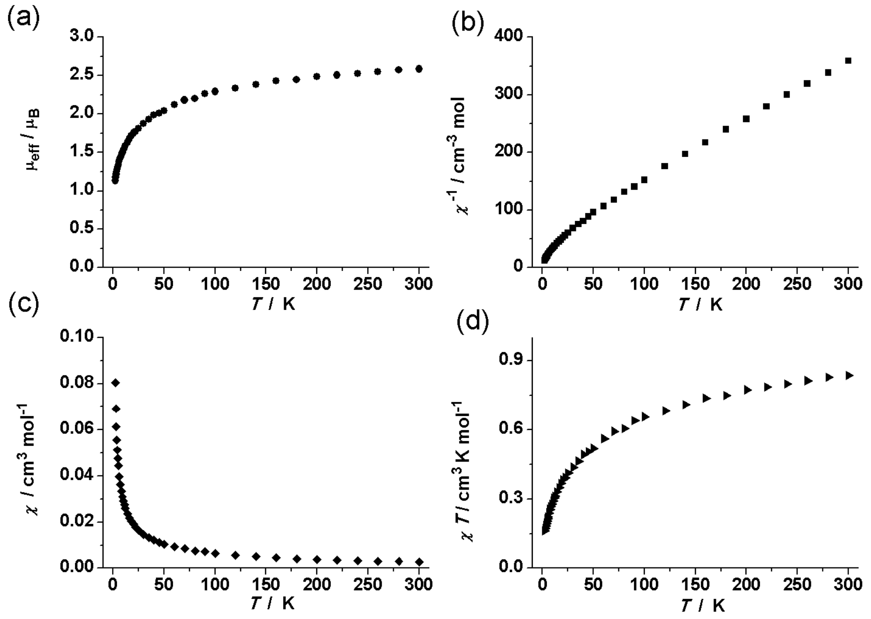

Figure S3. (a) μ_{eff} vs T, (b) 1/χ vs T, (c) χ vs T, and (d) χT vs T of [{U(Tren^{TIPS})(NCO)][K(B15C5)$_2$] (5).

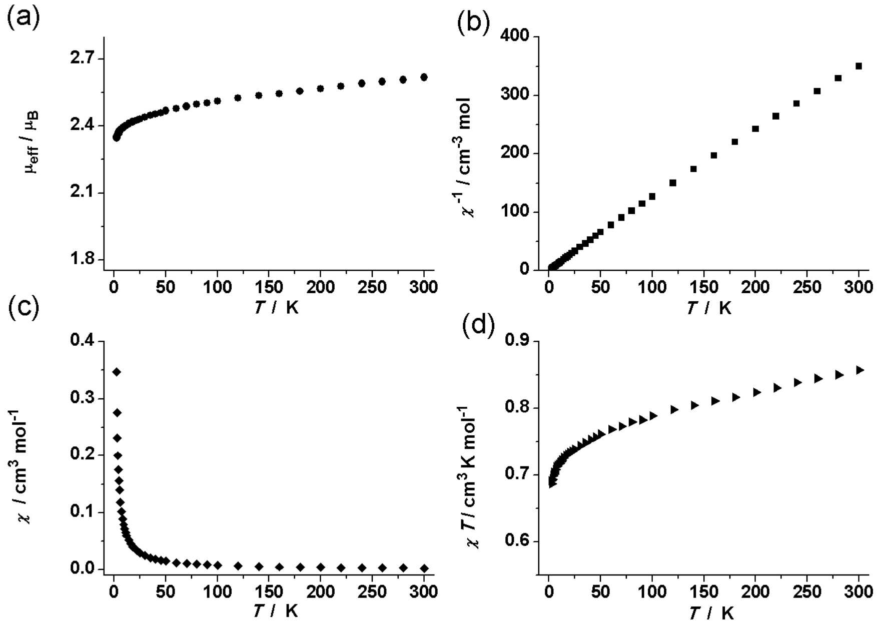

Figure S4. (a) μ_{eff} vs T, (b) 1/χ vs T, (c) χ vs T, and (d) χT vs T of [{U(Tren^{TIPS})(N)K}$_2$] (6).

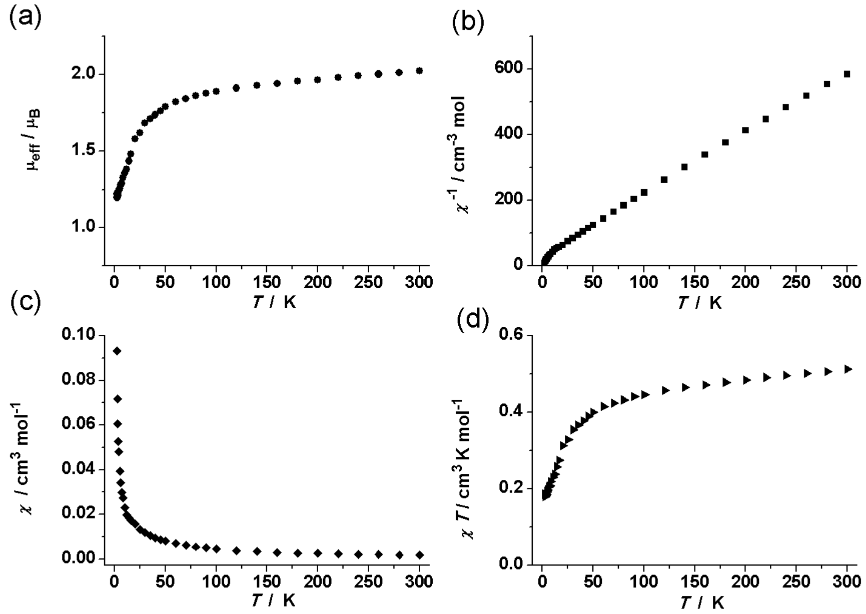

Figure S5. (a) μ_{eff} vs T, (b) 1/χ vs T, (c) χ vs T, and (d) χT vs T of [{U(Tren^{TIPS})(N)][K(B15C5)$_2$] (7).

EPR Data

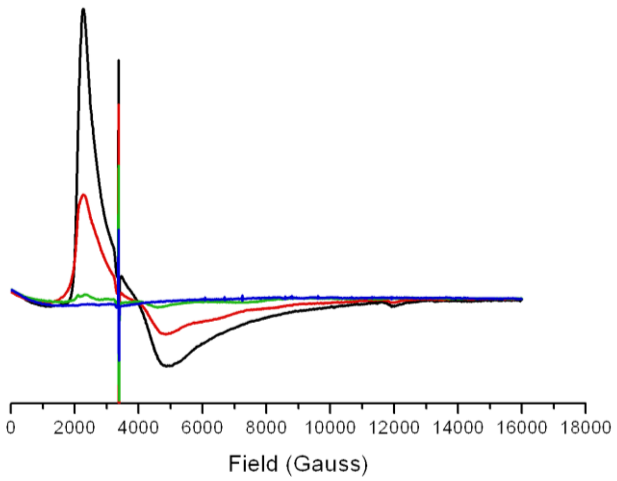

Figure S6. Solid state X-band EPR Spectra for [{U(Tren^{TIPS})(NCO)][K(B15C5)$_2$] (5) at 5 (black), 10 (red), 20 (green), and 40 K (blue).

X-ray Crystallography (CCDC numbers 1008236-1008239)

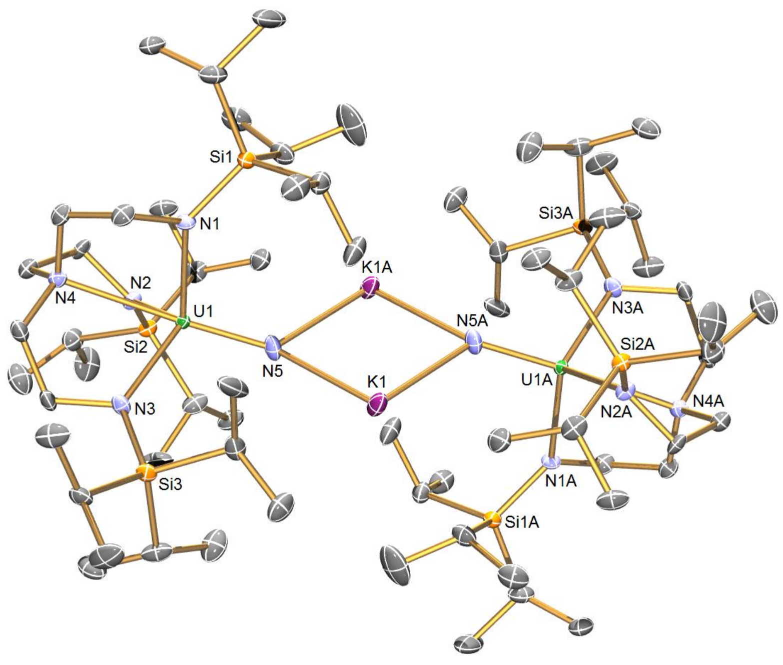

Figure S7. Molecular structure of [{U(Tren^{TIPS})(N)K}$_2$] (6); displacement ellipsoids set to 40%, hydrogen atoms omitted for clarity.

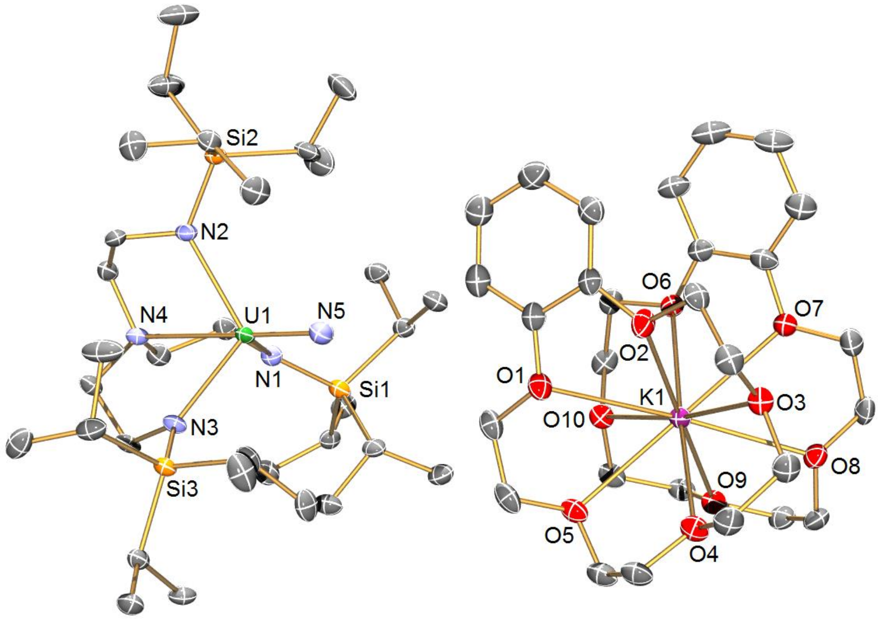

Figure S8. Molecular structure of [{U(Tren^{TIPS})(N)][K(B15C5)$_2$] (7); displacement ellipsoids set to 40%, hydrogen atoms omitted for clarity.

Computational Details

The assignment of *N*-bound cyanates in **3** and **5** was supported by DFT calculations. Unrestricted geometry optimizations were performed for the full models of **3** and the anion component of **5**, using coordinates derived from the X-ray crystal structures of **3** and **5** either as the *N*- or *O*-bound cyanate isomers. No constraints were imposed on the structures during the geometry optimizations. The calculations were performed using the Amsterdam Density Functional (ADF) suite version 2012.01.[4,5] The DFT geometry optimizations employed Slater type orbital (STO) triple-ζ-plus polarization all-electron basis sets (from the ZORA/TZP database of the ADF suite). Scalar relativistic approaches were used within the ZORA Hamiltonian for the inclusion of relativistic effects and the local density approximation (LDA) with the correlation potential due to Vosko et al[6]

was used in all of the calculations. Gradient corrections were performed using the functionals of Becke[7] and Perdew.[8]

All the structures involved in the reductive carbonylation reaction profile were fully optimized with the Becke's 3-parameter hybrid functional combined with the non-local correlation functional provided by Perdew/Wang (denoted as B3PW91).[9,10] The basis set used for uranium is the Stuttgart-Dresden small core RECP (relativistic effective core potential) in combination with its adapted basis set.[11,12] Si atoms were treated with the corresponding Stuttgart-Dresden RECP in combination with their adapted basis sets,[13] each one augmented by an extra set of polarization functions.[14,15] For the rest of the atoms the 6-31G(d,p) basis set was used.[16-18] In all computations no constraints were imposed on the geometry. All stationary points have been identified for minimum (number of imaginary frequencies Nimag=0) or transition states (Nimag=1). Intrinsic Reaction Paths (IRPs) were traced from the various transition structures to verify the reactant to product linkage.[19,20] GAUSSIAN09 program suite was used in all the calculations of the structures involved in the reductive carbonylation reaction profile.[21]

Table S1. Final coordinates and single point energy of 3 O-isomer after geometry optimization

1.C	1.628029	-0.002496	-6.173939
2.C	-0.632036	-0.628010	-5.202004
3.C	0.894345	-0.707022	-5.012557
4.C	3.679302	-2.139087	-3.552557
5.C	2.602758	2.560437	-3.555237
6.C	0.092225	2.369851	-3.341469
7.C	3.395353	-0.672146	-3.175298
8.C	1.444570	1.740123	-2.948835
9.C	-3.332822	0.512638	-2.586228
10.C	0.224497	-2.348750	-2.230692
11.C	3.997970	-0.346625	-1.797876
12.C	-4.812708	-2.369994	-1.153725
13.C	-4.259465	2.433368	-1.219599
14.C	-3.334781	1.198048	-1.205219
15.C	0.662894	-3.226330	-1.061807
16.C	-6.404396	-0.553606	-0.389124
17.C	-5.075623	-1.283936	-0.091431
18.C	-0.323830	3.425657	-0.027694
19.C	-1.320025	-3.216193	0.402291

20.C	-2.137501	-2.188034	1.179442
21.C	0.930397	-3.054673	1.382531
22.C	2.058337	-2.038498	1.518297
23.C	-4.107867	0.947766	1.837353
24.C	2.494060	2.824083	1.786040
25.C	4.846652	0.171681	1.956370
26.C	-2.986696	1.908116	2.271826
27.C	-4.518511	0.039991	3.014669
28.C	1.616622	2.076224	2.812516
29.C	4.020588	-0.047385	3.238424
30.C	1.524213	2.889217	4.121023
31.C	4.627151	0.734848	4.422949
32.C	-0.245140	-0.338051	4.603963
33.C	1.291081	-0.403049	4.671580
34.C	1.750781	-1.793745	5.152731
35.H	1.328821	-0.434021	-7.144258
36.H	1.386713	1.071307	-6.210321
37.H	-0.927545	-1.058812	-6.173653
38.H	2.721963	-0.093936	-6.097306
39.H	1.167920	-1.778448	-5.071247
40.H	-0.990293	0.410871	-5.183776
41.H	2.598004	2.513677	-4.654929
42.H	3.326000	-2.389402	-4.563469
43.H	-1.175439	-1.173546	-4.418890
44.H	-0.011524	2.423873	-4.436034
45.H	3.907250	-0.035900	-3.922095
46.H	4.761460	-2.351644	-3.521871
47.H	2.506115	3.621132	-3.273013
48.H	3.589128	2.217319	-3.213875
49.H	-3.007633	1.212663	-3.372018
50.H	0.690091	-2.758360	-3.142911
51.H	3.198659	-2.838817	-2.850009
52.H	0.009289	3.395631	-2.950149
53.H	-0.764888	1.799585	-2.958059
54.H	-4.340344	0.167161	-2.861508
55.H	-2.659886	-0.356103	-2.621519
56.H	-0.864568	-2.461704	-2.382919
57.H	-4.696870	-1.941211	-2.159003
58.H	-3.973544	3.114950	-2.037011
59.H	5.072843	-0.589811	-1.758640
60.H	1.539448	1.840659	-1.847266
61.H	-6.362473	-0.016552	-1.349405
62.H	3.893435	0.714613	-1.531853
63.H	-5.312042	2.152983	-1.379136
64.H	-5.655738	-3.080010	-1.198324
65.H	0.392490	-4.288778	-1.218937
66.H	3.502240	-0.929492	-1.005611
67.H	-3.905649	-2.953255	-0.942198
68.H	-7.239160	-1.272022	-0.456498
69.H	1.755687	-3.166437	-0.971432
70.H	-2.312127	1.588604	-1.032321
71.H	-1.771200	-3.337601	-0.591154
72.H	-4.203710	3.008402	-0.285486
73.H	-6.664301	0.179921	0.388438
74.H	-5.215921	-1.808777	0.873143
75.H	2.747620	-2.138636	0.659446
76.H	-1.313610	-4.206147	0.897723

77.H	2.624236	2.260057	0.850750
78.H	4.434770	-0.376589	1.098953
79.H	1.315856	-4.089506	1.298545
80.H	-3.164125	-2.577963	1.278523
81.H	-4.987637	1.561007	1.565181
82.H	2.046566	3.796129	1.525257
83.H	-2.716395	2.630930	1.490283
84.H	4.883429	1.234267	1.675449
85.H	5.887553	-0.163444	2.099381
86.H	3.496711	3.018972	2.194386
87.H	-1.753772	-2.116837	2.214608
88.H	0.302123	-3.000518	2.281805
89.H	2.656462	-2.327841	2.397888
90.H	-5.380298	-0.599279	2.774262
91.H	0.590333	2.041717	2.397485
92.H	-2.074659	1.348883	2.535481
93.H	-3.280212	2.484077	3.164850
94.H	-3.690685	-0.617449	3.323935
95.H	4.108846	-1.121486	3.492685
96.H	-4.794577	0.642844	3.895972
97.H	1.197423	3.918965	3.905526
98.H	4.601063	1.820930	4.244299
99.H	-0.636879	-1.027213	3.839083
100.H	5.684551	0.457334	4.574548
101.H	-0.610997	0.667276	4.352426
102.H	1.446409	-2.590369	4.454837
103.H	2.498228	2.955506	4.631126
104.H	0.806257	2.459742	4.833728
105.H	4.098869	0.541554	5.368966
106.H	2.841950	-1.857671	5.273569
107.H	1.616929	0.325925	5.437842
108.H	-0.703140	-0.622875	5.566134
109.H	1.298889	-2.039562	6.128922
110.N	0.581518	-0.929865	-1.992959
111.O	-0.215356	2.167543	0.021650
112.N	0.073293	-2.724590	0.210886
113.N	-2.090528	-0.880338	0.489567
114.N	1.525612	-0.657182	1.618199
115.N	-0.426187	4.599481	-0.072025
116.Si	1.539131	-0.151444	-3.277516
117.Si	-3.637286	-0.030889	0.246533
118.Si	2.118065	0.237662	3.045259
119.U	-0.015150	-0.098049	0.037225

Energy: −649.22422950 eV

Table S2. Final coordinates and single point energy of 3 N-isomer after geometry optimization

1.C	1.623431	-0.008925	-6.186612
2.C	-0.632326	-0.642862	-5.210670
3.C	0.894701	-0.715100	-5.023083
4.C	3.675504	-2.143161	-3.554346
5.C	2.633954	2.551040	-3.592869
6.C	0.118053	2.383540	-3.425250
7.C	3.394405	-0.674554	-3.181534
8.C	1.457202	1.750444	-2.996617
9.C	-3.340234	0.495871	-2.582735

10.C	0.223641	-2.330979	-2.229875
11.C	3.990339	-0.349135	-1.800938
12.C	-4.811958	-2.386863	-1.157336
13.C	-4.263475	2.421223	-1.221622
14.C	-3.346079	1.180962	-1.201600
15.C	0.665198	-3.213035	-1.063445
16.C	-6.410709	-0.570541	-0.406973
17.C	-5.085099	-1.301464	-0.096973
18.C	-0.295854	3.422781	-0.043592
19.C	-1.318439	-3.220271	0.402456
20.C	-2.141945	-2.197097	1.182030
21.C	0.932016	-3.048774	1.383614
22.C	2.058505	-2.029410	1.520066
23.C	-4.144284	0.929520	1.837758
24.C	2.552487	2.840996	1.838318
25.C	4.863794	0.156204	1.968107
26.C	-3.041006	1.905039	2.283613
27.C	-4.549002	0.014669	3.011638
28.C	1.655760	2.085687	2.841274
29.C	4.034190	-0.063310	3.247520
30.C	1.553495	2.881263	4.159603
31.C	4.642297	0.709783	4.437069
32.C	-0.231950	-0.339502	4.608851
33.C	1.304345	-0.403274	4.679076
34.C	1.763809	-1.794655	5.158752
35.H	1.326719	-0.443266	-7.156500
36.H	1.375861	1.063593	-6.224081
37.H	-0.928897	-1.080749	-6.178941
38.H	2.717951	-0.093630	-6.109876
39.H	1.173254	-1.785252	-5.080547
40.H	-0.994312	0.394831	-5.198101
41.H	2.647979	2.495001	-4.692056
42.H	3.327204	-2.393878	-4.567000
43.H	-1.172622	-1.184664	-4.422693
44.H	0.021762	2.400539	-4.521752
45.H	3.913519	-0.042126	-3.926597
46.H	4.756704	-2.359974	-3.516896
47.H	2.546374	3.616245	-3.322726
48.H	3.610803	2.200565	-3.231626
49.H	-3.003778	1.193848	-3.366114
50.H	0.685071	-2.743866	-3.142868
51.H	3.187913	-2.839884	-2.853567
52.H	0.047758	3.425919	-3.075354
53.H	-0.748696	1.842570	-3.022686
54.H	-4.347806	0.156861	-2.866616
55.H	-2.672240	-0.376776	-2.613141
56.H	-0.866100	-2.446047	-2.378442
57.H	-4.690538	-1.957737	-2.161747
58.H	-3.968207	3.103101	-2.036485
59.H	5.063763	-0.597795	-1.753911
60.H	1.524350	1.865866	-1.895377
61.H	-6.357146	-0.028931	-1.364453
62.H	3.889129	0.713247	-1.537833
63.H	-5.316918	2.149326	-1.390350
64.H	-5.652610	-3.099872	-1.207667
65.H	0.401080	-4.275788	-1.230930
66.H	3.485769	-0.926764	-1.010526

67.H	-3.904530	-2.966697	-0.939219
68.H	-7.245372	-1.287730	-0.487241
69.H	1.757780	-3.148187	-0.972359
70.H	-2.321717	1.565429	-1.021476
71.H	-1.768811	-3.340558	-0.591741
72.H	-4.213838	2.993510	-0.284918
73.H	-6.679024	0.160103	0.370544
74.H	-5.234050	-1.827027	0.865791
75.H	2.747215	-2.129235	0.660421
76.H	-1.311501	-4.211717	0.895784
77.H	2.669260	2.301737	0.887995
78.H	4.448977	-0.385430	1.107692
79.H	1.322865	-4.082252	1.302927
80.H	-3.166083	-2.594757	1.279531
81.H	-5.031063	1.530456	1.560371
82.H	2.128936	3.831068	1.604962
83.H	-2.772354	2.627125	1.501442
84.H	4.908224	1.219375	1.691670
85.H	5.902583	-0.186633	2.110561
86.H	3.558356	3.006519	2.254069
87.H	-1.759134	-2.129173	2.217960
88.H	0.303404	-2.995554	2.282958
89.H	2.657529	-2.323960	2.397490
90.H	-5.401142	-0.635659	2.765531
91.H	0.637925	2.064888	2.405814
92.H	-2.122004	1.359916	2.551897
93.H	-3.350703	2.476678	3.174259
94.H	-3.713868	-0.632301	3.323087
95.H	4.115820	-1.138896	3.497436
96.H	-4.837499	0.611898	3.893011
97.H	1.234472	3.916587	3.955542
98.H	4.620859	1.797340	4.263983
99.H	-0.621430	-1.025902	3.840118
100.H	5.698015	0.427603	4.590466
101.H	-0.599122	0.666322	4.360206
102.H	1.461986	-2.590411	4.458917
103.H	2.520865	2.936779	4.681738
104.H	0.824641	2.447052	4.858412
105.H	4.110142	0.514708	5.380467
106.H	2.855305	-1.857088	5.280988
107.H	1.628360	0.324273	5.447632
108.H	-0.691440	-0.629019	5.569196
109.H	1.311032	-2.042496	6.134047
110.N	0.580394	-0.910066	-2.001092
111.N	-0.214413	2.216648	-0.002957
112.N	0.073044	-2.726050	0.212641
113.N	-2.103276	-0.886007	0.499722
114.N	1.530986	-0.646387	1.625723
115.O	-0.380446	4.613560	-0.081990
116.Si	1.538822	-0.148025	-3.290764
117.Si	-3.649950	-0.046379	0.250271
118.Si	2.133746	0.236835	3.052428
119.U	-0.024200	-0.091471	0.042590

Energy: −649.89195974 eV

Table S3. Final coordinates and single point energy of the anion of 5 O-isomer after geometry optimization

1.C	0.578198	2.809479	-5.837267
2.C	0.200357	0.331024	-5.497791
3.C	0.795998	1.614085	-4.887329
4.C	-2.166212	3.488130	-3.402005
5.C	-2.459560	0.990517	-3.092229
6.C	2.700959	3.536426	-2.753782
7.C	-1.590008	2.214602	-2.750590
8.C	1.171066	3.621851	-2.589673
9.C	1.997896	-0.161237	-2.408199
10.C	1.692798	-1.662292	-2.397294
11.C	-4.469020	-2.260698	-1.932324
12.C	-1.069371	-2.779446	-1.302644
13.C	0.803450	4.127665	-1.183789
14.C	0.364286	-3.253397	-1.064186
15.C	-4.273649	-3.014126	-0.602868
16.C	-5.635413	-3.303087	0.061621
17.C	2.497584	-2.410738	-0.202311
18.C	-4.226275	0.400206	0.486238
19.C	-1.789554	2.863201	1.039562
20.C	3.438263	2.576924	1.081452
21.C	5.353112	-0.400481	1.460315
22.C	-2.328402	-4.946378	1.470831
23.C	2.227161	-2.074588	1.264163
24.C	-3.601995	-0.615704	1.463796
25.C	-2.694165	-3.542859	1.991179
26.C	2.529686	2.125868	2.241339
27.C	-4.545176	-0.844521	2.662911
28.C	4.578664	-0.305987	2.789258
29.C	-1.658004	-3.080210	3.030446
30.C	2.682784	3.084798	3.439366
31.C	5.382128	0.522513	3.813098
32.C	1.962834	-0.046070	4.410110
33.C	0.464449	0.306314	4.443643
34.C	2.178690	-1.480053	4.933177
35.H	0.975062	2.588638	-6.845581
36.H	0.670499	0.095003	-6.470004
37.H	-0.490741	3.046859	-5.955767
38.H	-0.880100	0.438399	-5.678875
39.H	1.078581	3.721433	-5.480152
40.H	0.331920	-0.540747	-4.841509
41.H	1.892264	1.462055	-4.835417
42.H	-2.181996	3.410691	-4.501263
43.H	-2.458290	0.775569	-4.172343
44.H	2.999601	3.278775	-3.781731
45.H	-1.592295	4.387963	-3.139453
46.H	-3.204447	3.655601	-3.070425
47.H	0.796801	4.365187	-3.319708
48.H	2.362384	0.084238	-3.422022
49.H	0.838281	-1.840010	-3.064821
50.H	-5.121503	-2.830993	-2.618464
51.H	-3.508031	1.161561	-2.795810
52.H	2.551412	-2.258218	-2.775014
53.H	3.184275	4.496834	-2.499988

54.H	-2.113461	0.087303	-2.568175
55.H	-3.516028	-2.081057	-2.446809
56.H	3.126298	2.774561	-2.083017
57.H	-1.131114	-2.344885	-2.320653
58.H	-4.939217	-1.279215	-1.775132
59.H	0.676747	-4.001559	-1.825363
60.H	2.860298	0.027389	-1.737959
61.H	-1.669570	2.365599	-1.655377
62.H	-1.697166	-3.689850	-1.343812
63.H	-0.272364	4.322006	-1.070239
64.H	-6.297625	-3.871268	-0.617252
65.H	-3.833894	-3.998044	-0.858993
66.H	1.334827	5.066620	-0.945815
67.H	3.320269	-1.772001	-0.550989
68.H	1.081216	3.392709	-0.412334
69.H	2.808940	-3.469204	-0.333128
70.H	-3.587482	0.572352	-0.391088
71.H	0.403406	-3.739687	-0.079009
72.H	-6.162120	-2.369785	0.317548
73.H	-5.205423	0.048480	0.122089
74.H	3.323887	1.928846	0.201205
75.H	-5.534226	-3.888455	0.988557
76.H	-3.088733	-5.353932	0.787453
77.H	3.198054	3.605391	0.767549
78.H	4.839840	-1.037255	0.727587
79.H	5.484329	0.586626	0.993431
80.H	-4.380587	1.376356	0.972182
81.H	-1.368749	-4.934618	0.929175
82.H	4.499815	2.565348	1.375165
83.H	6.362013	-0.823771	1.619962
84.H	1.430645	-2.748604	1.639570
85.H	3.130820	-2.361889	1.833649
86.H	-2.682271	-0.146575	1.869551
87.H	-2.216301	-5.663893	2.304152
88.H	1.482961	2.223039	1.889827
89.H	-5.488118	-1.323129	2.353813
90.H	-3.670877	-3.627543	2.506435
91.H	-0.674298	-2.920964	2.561306
92.H	-4.808869	0.118063	3.133132
93.H	4.522651	-1.335049	3.194679
94.H	2.489805	4.125501	3.126735
95.H	5.515944	1.561537	3.471853
96.H	-4.094534	-1.477800	3.440697
97.H	6.392820	0.099496	3.961254
98.H	-1.939166	-2.129962	3.505530
99.H	3.700538	3.055192	3.860566
100.H	-1.527643	-3.827038	3.834709
101.H	-0.099291	-0.332468	3.745016
102.H	1.981470	2.853232	4.253113
103.H	0.269186	1.347581	4.150823
104.H	1.695862	-2.218906	4.274550
105.H	4.892681	0.560309	4.797959
106.H	3.244422	-1.745801	5.006393
107.H	2.490589	0.644315	5.096663
108.H	0.037174	0.157039	5.451613
109.H	1.738530	-1.608274	5.938579
110.N	0.813265	0.619125	-2.012406

111.N	1.309986	-2.113587	-1.036886
112.N	-1.503923	-1.813142	-0.280179
113.O	-1.019413	1.867103	1.077673
114.N	1.867397	-0.654964	1.412403
115.N	-2.509681	3.801396	1.013123
116.Si	0.299089	1.951522	-3.030015
117.Si	-2.964355	-2.214560	0.596464
118.Si	2.710834	0.248354	2.646718
119.U	-0.001576	-0.018869	0.092256

Energy: −650.80652012 eV

Table S4. Final coordinates and single point energy of the anion of 5 N-isomer after geometry optimization

1.C	0.586102	2.788919	-5.872994
2.C	0.213922	0.312195	-5.518507
3.C	0.808660	1.599632	-4.915932
4.C	-2.154503	3.470503	-3.428631
5.C	-2.441154	0.975066	-3.104786
6.C	2.713221	3.534127	-2.780042
7.C	-1.572386	2.202328	-2.772526
8.C	1.181549	3.622246	-2.635043
9.C	2.004991	-0.183170	-2.433180
10.C	1.688889	-1.683022	-2.407524
11.C	-4.467124	-2.283518	-1.941566
12.C	-1.071342	-2.791709	-1.298039
13.C	0.800342	4.160130	-1.244672
14.C	0.363276	-3.265053	-1.061459
15.C	-4.273897	-3.029840	-0.607776
16.C	-5.636572	-3.320631	0.053368
17.C	2.496232	-2.408725	-0.205928
18.C	-4.250444	0.391072	0.470557
19.C	-1.767085	2.856901	1.204916
20.C	3.395280	2.587022	1.076593
21.C	5.342236	-0.377848	1.469504
22.C	-2.336925	-4.945000	1.481653
23.C	2.227563	-2.069376	1.261676
24.C	-3.622146	-0.620164	1.449415
25.C	-2.706093	-3.539957	1.995515
26.C	2.505823	2.131552	2.248779
27.C	-4.565704	-0.849280	2.648167
28.C	4.566956	-0.292170	2.798679
29.C	-1.672144	-3.073334	3.035259
30.C	2.674189	3.090893	3.444156
31.C	5.367436	0.534007	3.826645
32.C	1.948626	-0.038907	4.415979
33.C	0.453843	0.328135	4.456989
34.C	2.152042	-1.477602	4.930604
35.H	0.976382	2.560678	-6.882313
36.H	0.682425	0.071864	-6.490531
37.H	-0.483386	3.026936	-5.987178
38.H	-0.867104	0.417337	-5.698487
39.H	1.090018	3.703024	-5.525875
40.H	0.347689	-0.555639	-4.857656
41.H	1.905337	1.449530	-4.866183

42.H	-2.173955	3.385963	-4.527339
43.H	-2.439175	0.751734	-4.183209
44.H	3.022319	3.248159	-3.797413
45.H	-1.581736	4.374493	-3.176640
46.H	-3.194112	3.637419	-3.098386
47.H	0.816475	4.350356	-3.385097
48.H	2.364937	0.047393	-3.452572
49.H	0.830840	-1.859737	-3.070998
50.H	-5.112305	-2.860267	-2.629474
51.H	-3.490552	1.145995	-2.810506
52.H	2.543256	-2.284950	-2.787480
53.H	3.193301	4.501903	-2.548302
54.H	-2.093468	0.077307	-2.572490
55.H	-3.511745	-2.100879	-2.450961
56.H	3.130952	2.791023	-2.084001
57.H	-1.133066	-2.360873	-2.317843
58.H	-4.943887	-1.303741	-1.791771
59.H	0.670733	-4.019606	-1.819118
60.H	2.875647	0.003967	-1.772937
61.H	-1.639633	2.351261	-1.676132
62.H	-1.697617	-3.703732	-1.337756
63.H	-0.274656	4.373716	-1.160630
64.H	-6.296886	-3.889553	-0.627233
65.H	-3.830236	-4.013461	-0.858444
66.H	1.342255	5.095639	-1.016024
67.H	3.314466	-1.765688	-0.557667
68.H	1.044396	3.435447	-0.453376
69.H	2.817455	-3.465760	-0.329748
70.H	-3.602406	0.579658	-0.396051
71.H	0.405062	-3.744918	-0.073189
72.H	-6.165167	-2.388081	0.309120
73.H	-5.222000	0.031338	0.093668
74.H	3.271351	1.938212	0.197825
75.H	-5.536425	-3.906450	0.980221
76.H	-3.095972	-5.357300	0.799594
77.H	3.144282	3.614366	0.765925
78.H	4.831043	-1.012465	0.733485
79.H	5.469654	0.612183	1.007754
80.H	-4.426587	1.360063	0.966004
81.H	-1.377049	-4.932956	0.940444
82.H	4.461337	2.582319	1.355049
83.H	6.352817	-0.798025	1.627321
84.H	1.433807	-2.745563	1.639362
85.H	3.134420	-2.355658	1.827273
86.H	-2.705599	-0.141730	1.850236
87.H	-2.223506	-5.658642	2.318241
88.H	1.453027	2.222060	1.912338
89.H	-5.506990	-1.333138	2.341416
90.H	-3.684100	-3.623969	2.508625
91.H	-0.688209	-2.914804	2.566761
92.H	-4.833831	0.114081	3.115638
93.H	4.513752	-1.323446	3.199330
94.H	2.472771	4.131231	3.134463
95.H	5.499770	1.574469	3.488341
96.H	-4.112449	-1.477127	3.428848
97.H	6.379117	0.112827	3.974660
98.H	-1.953572	-2.121048	3.506307

99.H	3.698681	3.065928	3.849305
100.H	-1.543667	-3.817603	3.842300
101.H	-0.120602	-0.302985	3.759903
102.H	1.986843	2.857000	4.269127
103.H	0.267165	1.371242	4.165206
104.H	1.662579	-2.207744	4.267441
105.H	4.876636	0.568387	4.810882
106.H	3.215585	-1.752987	5.002284
107.H	2.485635	0.642873	5.104106
108.H	0.030707	0.181001	5.467305
109.H	1.710608	-1.607675	5.935362
110.N	0.832378	0.613791	-2.037267
111.N	1.308819	-2.126501	-1.045062
112.N	-1.506382	-1.822465	-0.278978
113.N	-1.064441	1.888935	1.051517
114.N	1.864676	-0.651756	1.415256
115.O	-2.466810	3.824810	1.363532
116.Si	0.318401	1.939425	-3.055887
117.Si	-2.968876	-2.219001	0.591118
118.Si	2.696906	0.255395	2.652341
119.U	0.004443	-0.016518	0.079728

Energy: −651.34451501 eV

Table S5. Gibbs free energies in a.u., along with Cartesian coordinates of the optimized structures

2 (singlet)

G= -2063.933984 au

U	0.11186900	-0.01471600	0.04824700
Si	2.17853800	-0.93103600	-2.81888900
Si	-0.73391900	3.50254800	0.60758700
Si	-0.94348800	-2.48162900	2.53418700
N	0.59793800	-0.36010700	-2.15852200
N	-1.99387500	-0.37430100	-1.25265800
N	-1.24817800	1.81964900	0.20789900
N	-1.02791600	-1.82162200	0.85758700
N	1.59571200	0.23458700	0.91872300
C	3.54281600	-0.94707900	-5.38470300
C	2.30816800	1.18191000	-4.85464500
C	2.33646100	-0.33988300	-4.65374700
C	1.22220100	-3.51962700	-3.79926000
C	-0.45827600	-0.31828800	-3.17363400
C	2.23203300	-2.86182600	-2.85064000
C	-1.75698300	0.21396200	-2.58245700
C	4.93664800	-1.07709700	-1.92035400
C	-1.63513700	4.83596000	-1.85975500
C	0.69889100	3.88640100	-1.85776200
C	3.82691400	1.16938700	-1.66094000
C	3.60683200	-0.34854200	-1.67479800
C	2.11089900	-3.45728700	-1.44329800
C	-2.19391400	-1.83300500	-1.30819000

C	-0.40192900	4.52273500	-1.00290700
C	-3.06078300	0.31663300	-0.50700000
C	-2.65122900	1.75338000	-0.20992900
C	-2.11319100	-2.43237200	0.09000600
C	-2.17550600	4.39441600	1.53969000
C	-1.86352200	5.86644900	1.84677900
C	1.68975900	4.76813300	1.60441700
C	-0.17801400	-5.15050900	1.59931600
C	0.91690500	3.44100300	1.59019200
C	-2.54471000	-4.96117000	2.40902100
C	-1.11652400	-4.41164900	2.56428600
C	1.96156700	-2.61736300	2.74758400
C	-2.67917200	3.68486500	2.80283400
C	0.76835300	2.90068300	3.01871900
C	0.71768800	-1.93383000	3.33209700
C	-2.44593000	-1.73956800	3.50163500
C	-2.34880300	-0.21483800	3.63154000
C	-2.75931600	-2.36733200	4.86896400
C	0.73968500	-2.05573000	4.86271300
H	3.52530300	-0.68101000	-6.45023100
H	2.18719000	1.43351400	-5.91703700
H	3.56659900	-2.03975900	-5.32294700
H	4.48919000	-0.57332500	-4.97949600
H	1.43731900	-0.74911900	-5.13985800
H	1.28121900	-3.12557300	-4.81960700
H	1.49793900	1.67083600	-4.30391600
H	3.24254800	1.64456900	-4.52188700
H	1.38542900	-4.60427800	-3.85802900
H	-0.20850300	0.34126900	-4.01701900
H	-0.64049900	-1.30610400	-3.62721900
H	3.23809100	-3.10387000	-3.22645500
H	0.19278100	-3.37696300	-3.44924200
H	-2.61313100	0.02178400	-3.24931600
H	5.35237100	-0.85181400	-2.90869100
H	-1.37911300	5.52513400	-2.67596000
H	0.91250100	4.48903900	-2.75090700
H	4.29723100	1.51809500	-2.58714400
H	-1.66933900	1.29454400	-2.44552700
H	-2.04332400	3.93340900	-2.33028000
H	4.84450100	-2.16495300	-1.84260500
H	0.40875500	2.88599100	-2.20216700
H	-1.38659400	-2.24532400	-1.91834700
H	-3.15029700	-2.07598900	-1.79878100
H	2.18201900	-4.55314000	-1.46499800
H	-2.44253800	5.30097200	-1.28425100
H	2.89113000	1.72068500	-1.53371800
H	5.68450900	-0.76217500	-1.18076400
H	1.63899200	3.77779300	-1.30742900
H	1.14525700	-3.20822600	-0.98474700
H	4.48952700	1.45414000	-0.83403000
H	-4.01171700	0.27464600	-1.06258900
H	2.89481700	-3.09384300	-0.77057800
H	-2.84972200	2.37946800	-1.09406900

H	-0.01739000	5.48521600	-0.63225600
H	3.26576200	-0.61855800	-0.66586500
H	-1.97508100	-3.51516300	-0.04318200
H	-3.19525500	-0.22025900	0.43513800
H	-1.54270500	6.42390800	0.96068100
H	-3.09025100	-2.32547700	0.59184300
H	1.92177800	5.13299800	0.59934700
H	-0.42632000	-4.93679600	0.55363400
H	-3.00294200	4.39056600	0.81297400
H	-3.34105100	2.12256800	0.56311900
H	1.53225900	2.71037400	1.04610100
H	-2.90835200	-4.85966300	1.38115000
H	-0.26310300	-6.23756100	1.73301500
H	2.64481200	4.64433200	2.13144500
H	-2.74821700	6.37674300	2.25090000
H	1.97494400	-2.60769000	1.65448900
H	0.87072900	-4.88245000	1.74041000
H	-1.07237300	5.95832300	2.59908400
H	1.14017200	5.56129900	2.12334700
H	-3.27154700	-4.46683800	3.05914900
H	-2.88116100	2.62222300	2.63882300
H	-2.56990300	-6.03305200	2.64679300
H	-2.16417700	0.27427400	2.66848000
H	2.87065300	-2.10489900	3.08541500
H	-3.60633100	4.14776800	3.16718100
H	2.03392700	-3.65881400	3.08171500
H	-3.30508500	-1.95616700	2.84625400
H	0.19855700	1.96728700	3.05320500
H	0.80412800	-0.86503600	3.08724900
H	-0.79163300	-4.65643300	3.58939700
H	-1.94886200	3.74829600	3.61493800
H	1.75303300	2.69501000	3.45652200
H	0.26699300	3.62469600	3.67103600
H	-3.27378900	0.20982100	4.04443000
H	-1.53571500	0.07937600	4.30527900
H	-2.81571300	-3.45949100	4.83705800
H	0.57228300	-3.08660900	5.19879400
H	-3.72223600	-2.00228700	5.25105600
H	1.71960200	-1.75014000	5.25229000
H	-0.00961700	-1.42333100	5.34588700
H	-2.00364900	-2.10662600	5.61582100

I (singlet)

G= -2177.18193 au

U	0.13855000	0.09621700	-0.20129000
Si	1.04608500	-1.93551100	-3.20303100
Si	-0.00821000	3.61752100	1.07296700
Si	-0.87343400	-2.07157300	2.63589000
N	0.00854500	-0.72020700	-2.37182100
N	-2.18929700	0.21962700	-1.06421100
N	-0.74336500	2.18343300	0.26616200
N	-1.18913900	-1.30868500	1.03073300
N	1.72378000	-0.08681000	0.51031000

C	2.58326200	-2.20839500	-5.63009000
C	2.15635300	0.17831200	-4.93411300
C	1.61121300	-1.25251600	-4.92159600
C	-0.82146500	-3.55277200	-4.86605800
C	-1.10870400	-0.29700800	-3.21734700
C	0.10322300	-3.57931400	-3.64008000
C	-2.03311600	0.65604200	-2.46334800
C	3.04112400	-3.82108000	-2.22193000
C	-1.62725900	5.88557200	0.06301800
C	0.17464100	5.01902400	-1.46906600
C	3.67039100	-1.39892800	-2.05958500
C	2.50220800	-2.39501600	-2.02600600
C	-0.61712000	-4.22522500	-2.45276400
C	-2.71533400	-1.15247400	-0.92889500
C	-0.24733400	5.21384500	-0.00802200
C	-2.93529200	1.19129600	-0.24511500
C	-2.13609200	2.47366200	-0.08554200
C	-2.50402700	-1.67389900	0.49083900
C	-0.94751400	3.97497600	2.72431200
C	-0.35320400	5.16885800	3.48586500
C	2.71267100	4.64056500	1.22374600
C	0.77793300	-4.13752700	1.43356500
C	1.88783800	3.34814100	1.33333300
C	-1.51151900	-4.88560400	2.10135300
C	-0.35729100	-3.92979200	2.44277600
C	1.93190000	-1.60746700	3.39912700
C	-1.12313000	2.76148600	3.64215000
C	2.25811600	2.60939500	2.62684700
C	0.50018300	-1.08484200	3.57961100
C	-2.55996600	-2.09372500	3.59936100
C	-3.20031400	-0.71672400	3.81591100
C	-2.55382000	-2.88955300	4.91411100
C	0.22795500	-0.92559100	5.08302500
H	2.79543900	-1.86341600	-6.65100200
H	2.37988000	0.49940800	-5.96065300
H	2.19145200	-3.22816100	-5.70744400
H	3.54426600	-2.26518400	-5.10697600
H	0.68323300	-1.24440500	-5.51394100
H	-0.32402600	-3.17547200	-5.76450400
H	1.44092600	0.89335000	-4.51844000
H	3.08275500	0.26989600	-4.35943900
H	-1.18051700	-4.56490700	-5.09715100
H	-0.78287200	0.22365700	-4.13207500
H	-1.69860400	-1.15729300	-3.57041100
H	0.94245200	-4.24267400	-3.90149200
H	-1.71183300	-2.93514600	-4.70080800
H	-3.01308000	0.74202800	-2.95886600
H	3.46779000	-3.96445600	-3.22173700
H	-1.61401500	6.84307100	-0.47537900
H	0.08228000	5.95547700	-2.03569800
H	4.18160700	-1.40967300	-3.02876200
H	-1.58795500	1.65650200	-2.43857500
H	-2.41112200	5.27582700	-0.39882000

H	2.27593500	-4.58859500	-2.07535400
H	-0.45366700	4.27448900	-1.97292400
H	-2.15577900	-1.77746700	-1.62424000
H	-3.78094000	-1.18642200	-1.20777400
H	-0.94477500	-5.24502600	-2.69586400
H	-1.93945500	6.09802300	1.09015600
H	3.35392800	-0.37476400	-1.85090100
H	3.84383600	-4.02250200	-1.50098100
H	1.21212500	4.68486500	-1.56220100
H	-1.51577800	-3.66347900	-2.17431400
H	4.41451700	-1.65967400	-1.29660500
H	-3.92853300	1.38012100	-0.68402300
H	0.01291600	-4.28804200	-1.56140700
H	-2.20880800	3.07244500	-1.00857900
H	0.46374700	5.92303400	0.44128700
H	2.07557800	-2.34656900	-1.01266100
H	-2.65935700	-2.76288400	0.43894500
H	-3.07452500	0.74184000	0.74016700
H	-0.27058100	6.06536000	2.86182200
H	-3.32066300	-1.30068000	1.12760700
H	2.60006400	5.13818700	0.25573600
H	0.48223900	-3.76973900	0.44538000
H	-1.95628800	4.27785200	2.40338800
H	-2.65613800	3.06999500	0.68042700
H	2.19877000	2.69205600	0.50968400
H	-1.88542400	-4.71104400	1.08568100
H	1.02032000	-5.20404500	1.32985200
H	3.78015700	4.41889600	1.35248700
H	-0.97565400	5.43166800	4.35182300
H	2.24694500	-1.59219600	2.35429700
H	1.69521700	-3.61659500	1.71293600
H	0.64785800	4.94558400	3.87016700
H	2.44303700	5.36562500	2.00068100
H	-2.36185400	-4.80244500	2.78442200
H	-1.54713800	1.90697300	3.10621100
H	-1.16987300	-5.92914600	2.13251600
H	-3.21422600	-0.11341600	2.90227900
H	2.63449100	-0.97384200	3.95689000
H	-1.79383600	2.99722100	4.47967500
H	2.03908200	-2.62541400	3.79255200
H	-3.21849900	-2.64730800	2.91278800
H	1.76869800	1.63588500	2.70261000
H	0.45845400	-0.08148500	3.13196300
H	0.02031200	-4.21098000	3.43955700
H	-0.17169200	2.43664700	4.07402000
H	3.33932100	2.42356200	2.65867100
H	2.00492200	3.19542500	3.51688200
H	-4.23714700	-0.81253900	4.16643100
H	-2.66292700	-0.13309600	4.57011200
H	-2.09413700	-3.87726100	4.80792700
H	0.26462000	-1.89018900	5.60270500
H	-3.57940000	-3.04566400	5.27557100
H	1.00120300	-0.29297800	5.53859200

H	-0.73840000	-0.46476500	5.30108500
H	-2.01452200	-2.36323000	5.70635900
C	1.57877100	1.69143500	-1.71231300
O	2.28472000	2.32697600	-2.33985500

$^{VI}TS_{I-II}$ (singlet)

G= -2177.166955 au

U	0.16329200	0.25017300	-0.19467500
Si	1.22792700	-1.65029000	-3.21985500
Si	-0.16496800	3.78525800	0.94986800
Si	-0.46008000	-2.10325200	2.58632700
N	0.06598300	-0.61286500	-2.29735700
N	-2.20408300	0.18904100	-0.98990700
N	-0.86922800	2.24165800	0.31734500
N	-0.93309300	-1.29389600	1.04197000
N	1.94178000	0.23172100	0.28997900
C	2.90047000	-1.44727200	-5.57370900
C	2.37825000	0.75760100	-4.44943400
C	1.87511200	-0.65886600	-4.74358100
C	-0.39814700	-3.00878500	-5.31638500
C	-1.09749400	-0.30741400	-3.13396400
C	0.33469100	-3.20238700	-3.98016800
C	-2.09131200	0.58822700	-2.40375600
C	2.98455200	-3.76862500	-2.25576100
C	-1.22181000	5.83277100	-0.89876100
C	0.81053200	4.57313800	-1.67581400
C	3.89548000	-1.45469200	-2.01282200
C	2.61002900	-2.29475800	-2.02835500
C	-0.56334300	-3.94727700	-2.98590700
C	-2.62467000	-1.21042200	-0.78288700
C	0.05646900	5.10705000	-0.45435900
C	-3.00205200	1.14147000	-0.20060000
C	-2.26468900	2.46191100	-0.07976600
C	-2.29273800	-1.66987800	0.63522300
C	-1.40457100	4.59153200	2.20125800
C	-0.82550700	5.86203100	2.84183200
C	2.48822600	4.63805400	1.78455300
C	0.05722200	-4.53008300	1.07502900
C	1.54448800	3.42659400	1.75602300
C	-1.85840800	-4.71372400	2.68030600
C	-0.50642700	-4.03320100	2.41295400
C	2.43404200	-2.34081000	2.37791700
C	-1.98528500	3.66513400	3.27552900
C	1.43501300	2.79895400	3.15304500
C	1.32056300	-1.54799600	3.07581700
C	-1.79198300	-1.58872500	3.88923500
C	-1.91741800	-0.06645400	4.00773700
C	-1.69633600	-2.23192200	5.28150700
C	1.56209300	-1.56070000	4.59264500
H	3.11587600	-0.93049100	-6.51840700
H	2.59007600	1.29491500	-5.38385500
H	2.55694800	-2.45632900	-5.82687700
H	3.85342100	-1.54986700	-5.04439900

H	0.97659700	-0.55354000	-5.37190900
H	0.24188500	-2.57144500	-6.08809000
H	1.64520800	1.34324000	-3.88689000
H	3.30198300	0.75095500	-3.86384600
H	-0.75361900	-3.97464500	-5.70050900
H	-0.82651000	0.20993300	-4.06796900
H	-1.61215100	-1.22775100	-3.44915800
H	1.18933600	-3.86200100	-4.19394000
H	-1.27889900	-2.36424900	-5.22089700
H	-3.07662100	0.58020700	-2.89644300
H	3.40998600	-3.92798600	-3.25391700
H	-0.97982400	6.62087600	-1.62458500
H	0.97264900	5.36967200	-2.41470900
H	4.42791200	-1.52668600	-2.96718400
H	-1.72551100	1.62031600	-2.41373000
H	-1.92929800	5.15800000	-1.39393400
H	2.13851100	-4.45231600	-2.14288900
H	0.24795500	3.77560300	-2.17573000
H	-2.06263800	-1.82626300	-1.48519400
H	-3.69954700	-1.32673000	-0.99521400
H	-0.84505000	-4.93738700	-3.36873000
H	-1.75024400	6.31238100	-0.06892300
H	3.71936500	-0.39954300	-1.80512900
H	3.75032300	-4.07681900	-1.53216000
H	1.78883600	4.16023700	-1.41889500
H	-1.49855100	-3.40293600	-2.80938900
H	4.57718900	-1.82472100	-1.23652800
H	-4.00165500	1.26716200	-0.64772500
H	-0.08544700	-4.09346500	-2.01279600
H	-2.33827400	3.01518000	-1.03019300
H	0.69757500	5.86757200	0.01816900
H	2.16612300	-2.22197800	-1.02461700
H	-2.44343100	-2.75998600	0.64041800
H	-3.12803500	0.71744700	0.79839700
H	-0.44498900	6.57148000	2.09921100
H	-3.04440200	-1.27071300	1.33587000
H	2.71267200	5.01290800	0.78134200
H	-0.58834300	-4.22765400	0.24353200
H	-2.24494100	4.91871300	1.57075400
H	-2.82522400	3.06910000	0.64511400
H	2.01852600	2.67583400	1.11510100
H	-2.59055900	-4.48899800	1.89620300
H	0.11564300	-5.62677600	1.05864700
H	3.44487000	4.36272400	2.24712400
H	-1.59089400	6.38560500	3.43035700
H	2.35914700	-2.29501000	1.28880300
H	1.05728400	-4.14345500	0.86646100
H	-0.00248200	5.62919900	3.52624800
H	2.07779700	5.47082200	2.36658900
H	-2.30639600	-4.42270100	3.63359900
H	-2.41058600	2.75041000	2.85164700
H	-1.74066400	-5.80555700	2.69444400
H	-1.98436400	0.41401200	3.02618800

H	3.41508400	-1.92797900	2.64421400
H	-2.78129200	4.17179500	3.83821100
H	2.43170800	-3.39413700	2.68263400
H	-2.72654800	-1.95951500	3.43898000
H	0.75648000	1.93966000	3.17416800
H	1.41671000	-0.50579700	2.74072200
H	0.18265100	-4.36565100	3.20659300
H	-1.22573500	3.35789500	4.00028000
H	2.41718300	2.45001500	3.49606600
H	1.07250900	3.52121300	3.89286300
H	-2.80336400	0.22163500	4.58927200
H	-1.04785700	0.36645900	4.51616200
H	-1.53974600	-3.31456000	5.24577700
H	1.40381100	-2.55474500	5.02800900
H	-2.61904100	-2.05548400	5.85030600
H	2.60008100	-1.27812100	4.81124200
H	0.91625200	-0.85715700	5.12497800
H	-0.87664700	-1.80753600	5.86719300
C	2.03439700	1.43463500	-0.96257600
O	3.06098000	1.92741900	-1.23883200

^{VI}II (singlet)

G= -2177.239227 au

U	-0.11577100	-0.05255900	0.05429900
Si	1.07750700	-1.32047000	-3.20979400
Si	-0.16949500	3.51471400	0.92442600
Si	-0.83828300	-2.49405800	2.69179100
N	-0.15700800	-0.51585700	-2.17250800
N	-2.51373300	-0.03575100	-0.74178700
N	-1.03841800	1.97960100	0.53688900
N	-1.26271500	-1.66354100	1.15356700
N	2.07270300	0.10311700	0.71502100
C	2.22226900	-1.00446600	-5.84872100
C	1.29185300	1.11194400	-4.83171900
C	1.15797400	-0.41368900	-4.91211200
C	-0.49875200	-3.40341700	-4.62391200
C	-1.40748000	-0.32762800	-2.92462200
C	0.60997400	-3.15964300	-3.59143600
C	-2.44549200	0.45323700	-2.12687400
C	3.68970100	-2.50225600	-2.69552800
C	-0.63088000	4.71971400	-1.67264400
C	1.51739600	3.44412900	-1.41357000
C	3.52257000	-0.02064700	-2.34526800
C	2.76083200	-1.35201000	-2.27577100
C	0.33118200	-3.94877500	-2.30794600
C	-2.97039900	-1.42966300	-0.63521300
C	0.48697600	4.32381700	-0.69924500
C	-3.23322200	0.87722600	0.15759900
C	-2.47855900	2.19119900	0.32240800
C	-2.62879500	-2.00616800	0.73493400
C	-1.40113400	4.77497300	1.72438000
C	-0.88450800	6.22171200	1.73422600
C	2.17397200	4.38282800	2.39031200

C	-0.70153000	-5.05695900	1.29020400
C	1.35385700	3.13569400	2.03135500
C	-2.59720100	-4.86342500	2.91825300
C	-1.16444300	-4.40443500	2.60063600
C	1.96873400	-2.98611200	2.14734900
C	-1.87911000	4.37254700	3.12633800
C	1.05416900	2.30899100	3.28670800
C	1.02405700	-2.16925500	3.04143100
C	-1.98456400	-1.77449500	4.07407600
C	-1.85890900	-0.25300500	4.20855800
C	-1.89205000	-2.44003400	5.45593100
C	1.42669900	-2.35257800	4.51177500
H	2.15831500	-0.55300700	-6.84785600
H	1.16120400	1.56903800	-5.82193600
H	2.11649600	-2.08741300	-5.97457700
H	3.23560100	-0.81397200	-5.47809400
H	0.18027600	-0.62875000	-5.37065100
H	-0.32577500	-2.86763200	-5.56266500
H	0.55367800	1.55993200	-4.15877100
H	2.27930200	1.40973000	-4.46712700
H	-0.57068700	-4.47180300	-4.86886100
H	-1.25389000	0.23082400	-3.86014400
H	-1.84209900	-1.29062400	-3.23340700
H	1.53630400	-3.56167200	-4.02941700
H	-1.48390400	-3.10174500	-4.25029000
H	-3.43377800	0.40920100	-2.61452600
H	3.95744200	-2.44491100	-3.75669400
H	-0.22569100	5.24240700	-2.54943900
H	1.89148000	3.92869900	-2.32530400
H	3.91253200	0.15912500	-3.35352000
H	-2.14324300	1.50377500	-2.08379400
H	-1.15658600	3.83345000	-2.04834000
H	3.24962700	-3.48817800	-2.51943900
H	1.07086600	2.49115300	-1.72968700
H	-2.44222500	-2.00431500	-1.39982800
H	-4.05131900	-1.51008900	-0.83974800
H	0.19068900	-5.01803200	-2.51556000
H	-1.37826300	5.37846600	-1.21712800
H	2.90034200	0.83532200	-2.07119300
H	4.62686300	-2.45893600	-2.12575900
H	2.38473100	3.21431800	-0.78701100
H	-0.57821100	-3.59062900	-1.81310400
H	4.38267600	-0.03067300	-1.66447400
H	-4.26189900	1.05544800	-0.19876600
H	1.14524100	-3.86261000	-1.57990200
H	-2.67198700	2.83586300	-0.55055100
H	0.99672900	5.24632500	-0.38185000
H	2.50039900	-1.52043100	-1.21988400
H	-2.78080200	-3.09239800	0.65578000
H	-3.29950800	0.38690100	1.13237500
H	-0.61445800	6.57610200	0.73468300
H	-3.37621600	-1.66314800	1.47013900
H	2.41771600	4.99375000	1.51498400

H	-1.29499600	-4.70811000	0.43800400
H	-2.27807700	4.76580100	1.06021700
H	-2.95654900	2.70469300	1.16808700
H	1.98447700	2.50889200	1.38812300
H	-3.30329700	-4.57053100	2.13343300
H	-0.81882700	-6.14808700	1.33840800
H	3.12288500	4.09626200	2.86172300
H	-1.65380000	6.90172300	2.12435300
H	1.71340300	-2.91504000	1.08492400
H	0.34481100	-4.84945700	1.05783600
H	-0.00297900	6.33767000	2.37270200
H	1.64459200	5.02294800	3.10468200
H	-2.97927900	-4.46348400	3.86081000
H	-2.20837900	3.32924300	3.18009000
H	-2.64047000	-5.95861700	2.98722800
H	-1.94015200	0.25558700	3.24155200
H	3.00278500	-2.63788500	2.25917500
H	-2.71581200	5.00373000	3.45465000
H	1.95386500	-4.04676600	2.42362400
H	-2.99482400	-1.98668000	3.68897800
H	0.47327200	1.41003000	3.05883300
H	1.18420300	-1.10839300	2.79763100
H	-0.52024900	-4.79463700	3.40641000
H	-1.07910900	4.49494500	3.86420400
H	1.98515000	1.98548500	3.76960000
H	0.48557700	2.88163700	4.02711500
H	-2.63603500	0.15586900	4.86839500
H	-0.89251600	0.02854000	4.64264400
H	-1.90075700	-3.53315900	5.40838100
H	1.20369900	-3.36235600	4.87776500
H	-2.73480000	-2.13409500	6.09010900
H	2.50701300	-2.19988700	4.63171100
H	0.92405400	-1.64091600	5.17190500
H	-0.97866600	-2.14892600	5.98229600
C	3.22952600	0.08101600	1.06015700
O	4.36531800	0.06284900	1.39882400

3 (triplet)

G= -2177.284979 au

U	-0.09535400	-0.06902300	0.08405400
Si	1.08066100	-1.30484500	-3.17861900
Si	-0.17132600	3.52009400	0.91632200
Si	-0.86067500	-2.52763600	2.67534900
N	-0.16425400	-0.50484400	-2.15044100
N	-2.50193200	-0.05251600	-0.73928400
N	-1.01708900	1.96402200	0.54558400
N	-1.27459100	-1.69667300	1.13359500
N	2.09667500	0.11548200	0.77946700
C	2.24758800	-0.99118000	-5.80975300
C	1.31350000	1.12716800	-4.80129900
C	1.17809400	-0.39836900	-4.88023500
C	-0.47640000	-3.38877400	-4.61161200
C	-1.40151800	-0.31045800	-2.92030100

C	0.62176400	-3.14474100	-3.56787200
C	-2.44498400	0.45338700	-2.11682400
C	3.69038900	-2.48538100	-2.64350400
C	-0.70777500	4.71374400	-1.67805600
C	1.47016000	3.48280900	-1.45071900
C	3.52102600	-0.00363800	-2.29777100
C	2.75835600	-1.33485200	-2.23120200
C	0.33199100	-3.93934100	-2.29023400
C	-2.97446300	-1.44042900	-0.65267700
C	0.43169600	4.34101700	-0.72115100
C	-3.21110600	0.85180500	0.17432900
C	-2.46034100	2.16727100	0.33818700
C	-2.63911600	-2.03765800	0.70892200
C	-1.41357300	4.75445800	1.73929500
C	-0.90047000	6.20206500	1.77615800
C	2.26896100	4.41131300	2.21115300
C	-0.74753200	-5.11018500	1.30521600
C	1.38722900	3.17498200	1.98190800
C	-2.65679800	-4.86335400	2.91100800
C	-1.21355200	-4.43208400	2.60170500
C	1.94125100	-3.02218200	2.09710400
C	-1.90114800	4.33185200	3.13237400
C	1.12610000	2.45084100	3.30808000
C	1.00666600	-2.21882200	3.01376900
C	-1.98116800	-1.77507900	4.05987000
C	-1.80314800	-0.25825200	4.19023600
C	-1.90413600	-2.44084000	5.44249600
C	1.42251400	-2.42945900	4.47689100
H	2.18890100	-0.54126600	-6.80990400
H	1.19029400	1.58239900	-5.79332300
H	2.14256800	-2.07428800	-5.93462900
H	3.25911500	-0.79990100	-5.43456500
H	0.20325400	-0.61200300	-5.34538300
H	-0.29566000	-2.85065700	-5.54750900
H	0.57102100	1.57724700	-4.13473900
H	2.29877400	1.42482500	-4.43068300
H	-0.54336900	-4.45682500	-4.85945500
H	-1.23799300	0.26157400	-3.84592300
H	-1.82911900	-1.27059900	-3.24793600
H	1.55347200	-3.54280500	-3.99788200
H	-1.46566700	-3.08996500	-4.24677700
H	-3.43437000	0.40585200	-2.60292500
H	3.96355200	-2.42938800	-3.70329600
H	-0.32477700	5.24391200	-2.56037200
H	1.81964100	3.97462200	-2.36828900
H	3.91567400	0.17409400	-3.30443300
H	-2.15004200	1.50494700	-2.06441300
H	-1.22141100	3.81756100	-2.04657300
H	3.24973200	-3.47117000	-2.46830500
H	1.03847700	2.52036500	-1.75741400
H	-2.45162500	-2.00995300	-1.42475000
H	-4.05660900	-1.50870400	-0.85775900
H	0.19986600	-5.00863500	-2.50313400

H	-1.46059200	5.35876900	-1.21204400
H	2.89905000	0.85438600	-2.02901700
H	4.62438100	-2.44044100	-2.06875700
H	2.35234900	3.27306600	-0.83748000
H	-0.58533300	-3.58755200	-1.80579000
H	4.37755500	-0.01332400	-1.61261800
H	-4.24515600	1.03279700	-0.16575800
H	1.13714900	-3.85198800	-1.55229300
H	-2.66124700	2.81335500	-0.53153900
H	0.92599200	5.27436900	-0.41236300
H	2.49574600	-1.50289600	-1.17531800
H	-2.78725400	-3.12317900	0.61586600
H	-3.26510700	0.35573700	1.14737900
H	-0.61732900	6.57203800	0.78590800
H	-3.38859400	-1.70599100	1.44738600
H	2.51030000	4.93798700	1.28227800
H	-1.32663700	-4.76502300	0.44167800
H	-2.28642200	4.75430000	1.06968400
H	-2.93539700	2.67640700	1.18783000
H	1.96489400	2.48130700	1.35747900
H	-3.34828400	-4.57619200	2.11140300
H	-0.88252800	-6.19847100	1.36804300
H	3.22020800	4.12110400	2.67586300
H	-1.67574700	6.87502400	2.16651700
H	1.67247300	-2.94366800	1.03812100
H	0.30441700	-4.92275400	1.08108700
H	-0.02789800	6.30933200	2.42854500
H	1.79304000	5.13135300	2.88604900
H	-3.04396700	-4.43756900	3.84002100
H	-2.21956800	3.28476100	3.17399200
H	-2.71715100	-5.95602500	3.00214600
H	-1.87732100	0.25148700	3.22298500
H	2.97593900	-2.67328100	2.19861800
H	-2.74838500	4.95074800	3.45694500
H	1.93157700	-4.08585000	2.36108000
H	-2.99933300	-1.95439200	3.67876000
H	0.48790900	1.57112100	3.18233900
H	1.17183400	-1.15397500	2.78875500
H	-0.58373500	-4.82094300	3.41937200
H	-1.11123200	4.45612400	3.88035400
H	2.06927100	2.11277900	3.75610300
H	0.63708100	3.10349200	4.03897100
H	-2.55970200	0.17823700	4.85607500
H	-0.82382900	-0.00882300	4.61536100
H	-1.95530600	-3.53289400	5.39763500
H	1.19362400	-3.44299600	4.82844500
H	-2.73002600	-2.10147400	6.08172700
H	2.50516600	-2.28813100	4.58867400
H	0.93288900	-1.72438000	5.15359100
H	-0.97666900	-2.18418900	5.96222600
C	3.25367300	0.08647600	1.12296600
O	4.39035600	0.06021500	1.46060700

7 (doublet)

G= -2063.990837 au

H	-2.92865000	-1.12207200	-3.16514200
H	-4.21082200	0.02446500	-2.79306200
H	-3.45573900	2.15693600	-1.10877300
H	-4.77576400	3.32166900	-0.97429400
C	-3.75293300	-0.91589900	-2.47207200
H	-4.49277100	-1.71116600	-2.60900600
C	-4.30880100	2.45443100	-0.49242600
H	-5.68460700	1.03946300	-1.32025000
H	-3.91356700	2.79420200	0.47089700
H	-2.49322800	-0.03311000	-0.97731000
H	-6.22970600	-2.46124500	-1.31506200
C	-5.33325300	1.32636500	-0.31579500
H	-7.04486100	2.63485600	-0.07180800
H	-7.18366100	-1.00048700	-1.02700200
C	-3.22435400	-0.85091500	-1.03066500
H	-1.59212300	-2.26467200	-1.30548800
H	2.44713800	3.87766600	-0.09115100
H	0.17251400	3.43830600	-0.85619000
C	-6.78754700	-1.90515400	-0.55511500
C	-6.56163500	1.80936300	0.46450100
C	-2.47783700	-2.13852500	-0.67198000
H	-7.64605400	-2.52490400	-0.26928000
H	-3.10230300	-3.02804500	-0.80385100
H	-1.31636000	-5.78399300	1.23192900
Si	-4.53245500	-0.32887300	0.25267700
H	-7.31736700	1.02923000	0.60032100
C	2.15041500	3.93859500	0.96342900
H	-6.29360000	2.19781700	1.45282500
H	-2.03948900	-4.23583100	1.65579200
C	-0.20048300	3.59594300	0.16367600
H	-0.56415500	-4.28835800	0.68049600
N	-0.99655400	0.59854100	0.87256100
H	2.02873800	5.00262200	1.19560700
H	-0.41961900	4.66491000	0.25980300
H	1.06746300	2.08815400	0.97577700
C	-1.08230900	-4.75329400	1.52455400
H	-2.13189900	-2.15544700	0.37173000
H	-4.82610800	-3.50024600	0.64473800
H	2.98979200	3.56506500	1.55637500
C	-5.91759400	-1.60331800	0.67418700
H	-1.13511300	3.03991300	0.25831500
C	0.85189400	3.14344700	1.18602000
C	-5.43027100	-2.89993500	1.33175000
H	2.99628900	1.89195900	3.09552900
H	-6.55349200	-1.07784700	1.40258900
H	-1.20414300	-6.53220000	3.60620200
N	-3.65463700	-0.08237900	1.85708600
H	0.35807500	-1.93727600	0.99941700
H	-6.28283900	-3.52060400	1.63307900
H	1.67226900	-4.00970200	0.70268700
H	-4.82519200	-2.71539300	2.22611100

H	2.58881400	-2.59706400	0.17142000
H	-2.02186900	-5.05357200	4.08943500
U	-1.22458600	-0.17394700	2.39638800
C	-0.26532200	-4.73725300	2.82288500
H	2.30509500	1.35952200	4.63345500
H	-2.27547900	4.73876500	1.65474700
C	-1.03441600	-5.49444700	3.91714200
C	2.74938500	2.21554100	4.11230600
H	0.67145900	-5.28767700	2.63861900
H	-5.54322400	0.40594600	2.72678600
C	2.14327200	-3.08509700	1.04563300
C	-4.46427100	0.50005300	2.90531600
H	3.69525800	2.44370500	4.61957300
C	1.15066500	-2.13913500	1.74777000
H	2.22349000	-0.33176000	1.15279300
H	-0.10303700	6.13563200	2.02614600
H	-4.25970100	1.58479700	2.92559200
Si	0.24482600	3.18165800	3.01546800
H	2.31930000	4.24548900	3.61246400
H	-0.50812100	-5.52610000	4.87347400
C	-2.33259400	4.55849600	2.73175500
C	1.80592800	3.42408900	4.13462600
H	-2.79049000	3.57202800	2.86889200
Si	0.36107700	-2.98631300	3.29479100
N	-1.00520000	-1.86243900	3.69900800
N	-0.60482400	1.63156500	3.50809000
H	2.96598300	-3.35887900	1.71583700
C	1.86662300	-0.81699400	2.06691700
H	-2.78534200	-2.77182100	4.40668300
H	-3.01788600	5.30663100	3.15004300
C	-0.29790600	6.00773800	3.09610300
C	-0.95198200	4.64879900	3.39365500
H	-4.37209500	-1.17885200	4.23052900
H	0.65313000	6.13968800	3.62168000
H	2.73390900	-0.99373300	2.71087100
C	-4.13931300	-0.11031500	4.26676400
H	1.27928900	-0.05534900	2.61421800
C	-1.89677100	-2.25232900	4.79397700
N	-2.71633300	0.04310000	4.61222700
H	-0.95511800	6.83092300	3.40319500
H	1.87945200	-5.19730800	4.90059500
H	-1.41424300	-2.95822700	5.48281600
H	-4.78059700	0.33661600	5.04094000
C	1.59355100	3.89992700	5.57904300
H	-1.11362700	4.60882900	4.48217800
H	2.54615400	4.22365000	6.01692300
H	-3.00573900	2.12528700	4.61961200
C	-0.93978000	1.69657000	4.94474700
H	2.62912000	-2.93948500	4.10679000
H	1.21244900	3.10061500	6.22397900
C	1.69498900	-3.01175500	4.68689300
C	-2.28855500	-1.00630700	5.56293900
C	-2.41118300	1.38385500	5.16010700

H	-0.31063200	1.01477300	5.53590800
H	0.90347500	4.74657300	5.65181500
C	1.80152600	-4.29785100	5.51847500
H	0.94320700	-4.42302600	6.18779400
H	-0.76063500	2.69140800	5.36873300
H	-3.07380800	-1.20409400	6.30671300
H	-1.41061600	-0.63796200	6.09831800
H	1.58223700	-0.84188300	5.06087500
H	-2.69633500	1.43619600	6.22228500
H	2.69488700	-4.26218800	6.15330900
C	1.64068500	-1.78632000	5.60987700
H	0.77483200	-1.83347600	6.28043000
H	2.53335400	-1.74389900	6.24501500

^{V}TS$_{I-II}$ (doublet)

G= -2177.245179 au

U	0.24687500	0.31676400	-0.26408800
Si	1.21248100	-1.77824500	-3.29820000
Si	-0.22038300	3.88072400	0.98450800
Si	-0.43171900	-2.05371800	2.59719100
N	0.07770200	-0.77405900	-2.39479500
N	-2.19543200	0.23209100	-1.03873100
N	-0.84736700	2.41108100	0.23838700
N	-0.92063900	-1.21995000	1.12036900
N	1.97885300	0.33986400	0.34787900
C	2.87843800	-1.62594800	-5.68965700
C	2.34520800	0.59091000	-4.59332900
C	1.85308400	-0.83390100	-4.86662100
C	-0.44663100	-3.20514200	-5.32374200
C	-1.13973700	-0.52671300	-3.15897700
C	0.36496000	-3.37987500	-4.03204000
C	-2.06424400	0.48242700	-2.48003600
C	3.14124600	-3.81070800	-2.46643700
C	-1.15285300	6.02250400	-0.82051900
C	0.98641500	4.87104900	-1.45481800
C	3.85104100	-1.44629600	-2.06163700
C	2.64554500	-2.39162700	-2.14803000
C	-0.45915100	-4.13647700	-2.98433800
C	-2.63745300	-1.12768100	-0.68922800
C	0.10473100	5.32226000	-0.28697800
C	-2.95952800	1.28491500	-0.35892600
C	-2.18417600	2.59725600	-0.31675000
C	-2.31523800	-1.47658500	0.76598300
C	-1.53168100	4.64245200	2.20928200
C	-0.98706200	5.84425400	2.99268800
C	2.39828200	4.68171400	2.01994000
C	-0.02353200	-4.47787800	1.05810800
C	1.44508300	3.48894500	1.86676700
C	-1.87726300	-4.65663700	2.72676700
C	-0.52222400	-3.99669500	2.42634600
C	2.45108600	-2.39482500	2.34600700
C	-2.19998300	3.63885100	3.15360800
C	1.26047100	2.77552600	3.21235900

C	1.37800300	-1.56388200	3.06088700
C	-1.70749900	-1.58955600	3.98975500
C	-1.85106800	-0.07290800	4.14933500
C	-1.55449400	-2.26210500	5.36151900
C	1.66388300	-1.54681100	4.56817800
H	3.11457800	-1.11284600	-6.63382900
H	2.53126400	1.13132200	-5.53372400
H	2.52551200	-2.63189400	-5.94531200
H	3.82371900	-1.74213200	-5.14720600
H	0.94976900	-0.73993800	-5.49098800
H	0.14318300	-2.75425600	-6.12837700
H	1.61670800	1.16229300	-4.01102800
H	3.27965500	0.60058200	-4.02376200
H	-0.80777900	-4.17762500	-5.69159500
H	-0.95408400	-0.11914900	-4.17085200
H	-1.71969800	-1.45143900	-3.33421700
H	1.22198500	-4.01936100	-4.29234000
H	-1.33007200	-2.57427500	-5.17647600
H	-3.05420400	0.49498200	-2.97169800
H	3.58227200	-3.86977900	-3.46993500
H	-0.88316400	6.84642800	-1.49844000
H	1.19708700	5.70477900	-2.14163200
H	4.36584300	-1.36068400	-3.02609000
H	-1.63304900	1.48472700	-2.58915900
H	-1.78743400	5.33725700	-1.39474100
H	2.35113000	-4.56556700	-2.40952400
H	0.49914500	4.07904700	-2.03418700
H	-2.08506700	-1.81990600	-1.32763600
H	-3.71757400	-1.25147500	-0.88938200
H	-0.81116200	-5.10498900	-3.37036100
H	-1.77027900	6.45060700	-0.02331500
H	3.57220100	-0.44758200	-1.72611400
H	3.92639000	-4.10682800	-1.75660200
H	1.94507800	4.46471700	-1.12267400
H	-1.34739600	-3.56637700	-2.68907200
H	4.58341200	-1.83111300	-1.33839900
H	-3.94621200	1.41563100	-0.84162300
H	0.10802500	-4.33170000	-2.06908900
H	-2.16951600	3.03570200	-1.33278900
H	0.66955700	6.07537700	0.28485300
H	2.18761500	-2.41586700	-1.14840600
H	-2.60224900	-2.53886500	0.86587900
H	-3.12827200	0.95679000	0.66991300
H	-0.54820000	6.60575600	2.33807200
H	-3.02108200	-0.93748700	1.42668500
H	2.66058800	5.13095000	1.05702000
H	-0.68168500	-4.12285900	0.25820900
H	-2.32315800	5.03274000	1.55090900
H	-2.81281800	3.29135800	0.26887400
H	1.93024200	2.76082400	1.20410500
H	-2.62944900	-4.39372400	1.97359400
H	-0.00165400	-5.57688500	1.00635100
H	3.33753000	4.36199300	2.49311800

H	-1.78104000	6.33334400	3.57722200
H	2.36918500	-2.30864400	1.25997200
H	0.98048100	-4.11450400	0.82746600
H	-0.21038700	5.54030000	3.70433000
H	1.97655100	5.47498000	2.64980700
H	-2.28680900	-4.37746000	3.70167300
H	-2.60976800	2.78216800	2.61052500
H	-1.78840900	-5.75343000	2.71105600
H	-1.96073900	0.42402400	3.18071300
H	3.45217000	-2.02974900	2.61316600
H	-3.02259100	4.10749500	3.71493400
H	2.40609000	-3.45586500	2.62457300
H	-2.65488900	-1.95790500	3.56324200
H	0.59745200	1.90952200	3.12518700
H	1.49149700	-0.53801700	2.68511100
H	0.18800000	-4.35993300	3.18781300
H	-1.49404500	3.23977000	3.88872400
H	2.22425700	2.41080700	3.59253100
H	0.84072400	3.44286900	3.97553800
H	-2.71800600	0.19196100	4.77243800
H	-0.96687300	0.36196500	4.63111700
H	-1.37430100	-3.34039900	5.29401800
H	1.51674900	-2.53085700	5.03231600
H	-2.45847500	-2.11987400	5.97246500
H	2.70939500	-1.26130600	4.75211400
H	1.03492000	-0.82995300	5.10471400
H	-0.72142200	-1.83403800	5.92693100
C	2.07823300	1.60480000	-1.14171600
O	3.11552300	2.09788200	-1.35957300

^{V}II (doublet)

G= -2177.309307 au

U	0.13572500	0.04206200	0.03389800
Si	1.38467600	-1.32503200	-3.23185500
Si	-0.01203500	3.74194400	0.64486900
Si	-0.89675300	-2.35023500	2.76155700
N	0.06844000	-0.87140200	-2.16314000
N	-2.38326200	0.08294500	-0.91117000
N	-0.70480200	2.27348900	-0.01702800
N	-1.26827100	-1.26931700	1.42895500
N	2.37497300	-0.00020800	0.89291800
C	2.28533400	-0.86208800	-5.96542600
C	0.76454200	0.91527700	-5.01785300
C	1.12268100	-0.57505700	-5.00655500
C	0.41863600	-3.85009700	-4.41447700
C	-1.24877700	-1.22406500	-2.68987500
C	1.50601800	-3.24673200	-3.51638500
C	-2.30275900	-0.17265200	-2.35476400
C	4.28520000	-1.57621500	-2.89917600
C	-0.58697700	5.75548100	-1.43940400
C	1.46403200	4.33471800	-1.74572500
C	3.33128600	0.72111000	-2.54089300
C	3.05416500	-0.78443600	-2.43594200

C	1.56254000	-3.99422400	-2.17990200
C	-3.11185300	-0.97919700	-0.20492000
C	0.54536100	5.00999800	-0.72236300
C	-2.94324600	1.40927300	-0.62221400
C	-1.90854900	2.51364700	-0.81042300
C	-2.70749800	-1.06258600	1.26360700
C	-1.32211100	4.69512100	1.72349500
C	-0.75340800	5.94075200	2.41393600
C	2.59804500	4.42213900	1.79555100
C	-1.41986900	-4.65872000	1.06814000
C	1.57377600	3.28849800	1.64482900
C	-3.05587000	-4.39857300	2.93641700
C	-1.60009600	-4.15568200	2.50635900
C	1.70882100	-3.27757200	1.86418100
C	-2.07041800	3.80751800	2.72371000
C	1.28754200	2.66206700	3.01624900
C	1.01752800	-2.46059200	2.96309500
C	-1.73639600	-1.65231300	4.37036800
C	-1.28678200	-0.21481200	4.65313900
C	-1.66489700	-2.50060400	5.64788100
C	1.49035000	-2.94906900	4.33798000
H	2.05272000	-0.53147700	-6.98882800
H	0.47458900	1.24779600	-6.02599400
H	2.53188400	-1.92876100	-6.01527100
H	3.19436700	-0.33128200	-5.65902100
H	0.24680500	-1.11519600	-5.39920000
H	0.39121500	-3.38639900	-5.40686300
H	-0.06289400	1.13767800	-4.33745200
H	1.61069100	1.53625400	-4.70485100
H	0.58307300	-4.92785300	-4.56392000
H	-1.27121900	-1.32185300	-3.79028800
H	-1.59429800	-2.20619000	-2.31720500
H	2.46858400	-3.40175800	-4.02827700
H	-0.57773600	-3.73930800	-3.97227400
H	-3.29372100	-0.46368100	-2.75489500
H	4.48099100	-1.44374500	-3.97029600
H	-0.18609000	6.49057500	-2.15349500
H	1.85598100	5.05648200	-2.47787400
H	3.57671400	1.01469800	-3.56934600
H	-2.01411300	0.76320000	-2.84277100
H	-1.22278300	5.07128700	-2.01262700
H	4.18663100	-2.65058700	-2.71462400
H	0.92363600	3.55986800	-2.30113000
H	-2.85897100	-1.93290000	-0.67769000
H	-4.20499000	-0.83557600	-0.30899000
H	1.69959900	-5.07601900	-2.32652700
H	-1.23461000	6.30091200	-0.74413700
H	2.47438200	1.32320600	-2.22159100
H	5.18223300	-1.23631800	-2.36343800
H	2.32285400	3.84589700	-1.27350300
H	0.63447700	-3.85184100	-1.61547000
H	4.18290600	1.00516800	-1.90863600
H	-3.84253900	1.59601500	-1.24102100

H	2.38101700	-3.64328700	-1.54204700
H	-1.68319000	2.61116600	-1.88905800
H	1.13725400	5.76595900	-0.18325100
H	2.91667900	-1.00054000	-1.36625500
H	-3.31873500	-1.87618600	1.69201600
H	-3.25917700	1.41778300	0.42538100
H	-0.25444700	6.61611400	1.70947800
H	-3.05835400	-0.15254400	1.78498200
H	2.94212100	4.80703500	0.83058700
H	-2.02298300	-4.06650000	0.37098700
H	-2.06845300	5.04905900	0.99496200
H	-2.42917600	3.45328500	-0.54991000
H	2.05011200	2.50446200	1.03794800
H	-3.75900100	-3.89273500	2.26407800
H	-1.73901300	-5.70749500	0.97116800
H	3.48502500	4.06601600	2.33743000
H	-1.54326200	6.51814800	2.91741200
H	1.40251400	-2.96926400	0.85927600
H	-0.38541200	-4.59394200	0.72452400
H	-0.01925600	5.66955500	3.18150400
H	2.19353000	5.26961900	2.36248800
H	-3.27267200	-4.05189500	3.95086600
H	-2.46587500	2.90669300	2.24480700
H	-3.30203900	-5.47041800	2.89667100
H	-1.39703400	0.41713100	3.76672100
H	2.79841600	-3.15675700	1.92087700
H	-2.91161200	4.34592700	3.18569500
H	1.49225700	-4.34857800	1.96588100
H	-2.79890600	-1.60305800	4.08112100
H	0.56138600	1.84466600	2.95838800
H	1.36036000	-1.42248600	2.84748000
H	-0.96369800	-4.77597300	3.15982900
H	-1.41601700	3.47415500	3.53586400
H	2.20593300	2.24955300	3.45422100
H	0.89353000	3.40425900	3.72177300
H	-1.86777300	0.23898200	5.46962300
H	-0.23112400	-0.17705900	4.94920200
H	-1.90766300	-3.55478600	5.47682500
H	1.09248200	-3.94255900	4.58322400
H	-2.36232200	-2.12348100	6.41055400
H	2.58625800	-3.02701800	4.36199200
H	1.19796200	-2.26766100	5.14193000
H	-0.66615500	-2.47239900	6.09412300
C	3.49771800	-0.02094800	1.32488500
O	4.61411700	-0.04267100	1.75500300

5 (quartet)

G= -2177.339747 au

C	-0.02556700	2.44358100	-6.07961600
C	-0.25517100	0.02870700	-5.40919300
C	0.42668400	1.36333100	-5.08839000
C	-1.85099100	3.85278500	-3.20149500
C	-2.51311800	1.43322300	-3.00834300

C	2.91282600	3.21350100	-3.59773700
C	-1.42933200	2.46942600	-2.68728700
C	1.47776100	3.47896200	-3.12530300
C	2.02532200	-0.15319500	-2.56295600
C	1.76691300	-1.65487400	-2.45067200
C	-4.42193800	-2.09453000	-1.91130700
C	-0.95271800	-2.75916400	-1.29442400
C	1.48868400	4.07015900	-1.71220700
C	0.49235900	-3.19003300	-1.04966400
C	-4.21723500	-2.93754900	-0.64791000
C	-5.56027000	-3.24373300	0.02696800
C	2.59723800	-2.25742400	-0.24411800
C	-4.18074200	0.33929800	0.68087000
C	-1.66251300	2.86587200	1.46804800
C	3.35205500	2.75419000	1.45131200
C	5.31902500	-0.13555100	1.78002800
C	-2.29147600	-5.04743900	1.24319100
C	2.31902900	-1.95364000	1.22572100
C	-3.48736600	-0.70847700	1.56190800
C	-2.59404900	-3.67798800	1.86438900
C	2.34516900	2.18639000	2.45940600
C	-4.36698100	-1.02517500	2.77922300
C	4.44042800	-0.18525200	3.03593500
C	-1.50259000	-3.30658800	2.87231300
C	2.32606400	3.05715900	3.72356500
C	5.10287600	0.59248300	4.18104600
C	1.70367800	-0.10958900	4.45309200
C	0.19566100	0.14668300	4.36755900
C	1.97201600	-1.53396200	4.95273500
H	0.18393000	2.14456100	-7.11764500
H	-0.01411300	-0.30686300	-6.42906600
H	-1.10558100	2.62074000	-6.01349800
H	-1.34568300	0.10824500	-5.34677500
H	0.47432500	3.40307800	-5.90928200
H	0.04675700	-0.76164500	-4.71666100
H	1.50558000	1.21740200	-5.25639300
H	-1.94240100	3.87068600	-4.29405200
H	-2.68808000	1.35974300	-4.08876400
H	2.95601000	2.86493100	-4.63622100
H	-1.14347700	4.63871800	-2.91976100
H	-2.83090400	4.13499400	-2.79211000
H	1.03583000	4.22616800	-3.80318900
H	2.34037000	0.03275400	-3.60433600
H	0.92204800	-1.89973700	-3.10063000
H	-5.08319300	-2.60686200	-2.62642700
H	-3.47113900	1.70846700	-2.54625700
H	2.63618000	-2.24275100	-2.80189000
H	3.52877200	4.12274300	-3.53285900
H	-2.24532200	0.43536200	-2.64507400
H	-3.47579300	-1.88758700	-2.41909900
H	3.39838300	2.45333800	-2.97487200
H	-1.02428800	-2.33715800	-2.31284900
H	-4.88197500	-1.12776400	-1.68272900

H	0.81478300	-3.95849700	-1.77871800
H	2.91146000	0.09677600	-1.95156700
H	-1.36350500	2.54111400	-1.59013100
H	-1.55178300	-3.68569000	-1.33462700
H	0.48926500	4.35448500	-1.36752300
H	-6.25040900	-3.74832700	-0.66571100
H	-3.80186800	-3.90331200	-0.97729500
H	2.12591300	4.96514700	-1.65421200
H	3.37543800	-1.56786000	-0.58712000
H	1.87727500	3.34381300	-0.99142100
H	2.98134900	-3.28687100	-0.37849100
H	-3.58913700	0.59289200	-0.20283400
H	0.54629300	-3.63928600	-0.05267200
H	-6.05942700	-2.32613800	0.35970500
H	-5.15914400	-0.01913000	0.33742500
H	3.40756000	2.14862700	0.54086000
H	-5.44756600	-3.89068800	0.90411600
H	-3.08311000	-5.38485600	0.56505000
H	3.07971300	3.77612700	1.15524200
H	4.87416300	-0.68533600	0.94565400
H	5.47695000	0.89206800	1.43802500
H	-4.34842400	1.27020300	1.23760400
H	-1.35558800	-5.03043700	0.67242300
H	4.36076700	2.80451600	1.88010600
H	6.31219500	-0.56787900	1.97424400
H	1.57347200	-2.67998700	1.59792700
H	3.24434500	-2.20248300	1.77313700
H	-2.56373700	-0.24171600	1.94057700
H	-2.17578400	-5.81910300	2.01897100
H	1.34585100	2.26469200	2.00285700
H	-5.30792900	-1.50628000	2.48527700
H	-3.54345000	-3.77222200	2.41471000
H	-0.55232400	-3.10872500	2.36310000
H	-4.63184800	-0.10243600	3.31371900
H	4.40463800	-1.23953100	3.35351800
H	2.15550800	4.11136700	3.46397400
H	5.20801200	1.65544600	3.93409400
H	-3.87292400	-1.68651700	3.49755600
H	6.11396600	0.21190000	4.38992500
H	-1.74996000	-2.40295500	3.43828700
H	3.27792000	3.00991100	4.26650700
H	-1.33003000	-4.11247000	3.60117400
H	-0.26531800	-0.52086400	3.63111000
H	1.53517300	2.76587500	4.42103500
H	-0.04165200	1.16973200	4.05839300
H	1.61179300	-2.28244400	4.23711800
H	4.53157100	0.52938700	5.11350900
H	3.03860900	-1.72221900	5.11871100
H	2.12552200	0.58808200	5.19377800
H	-0.30100500	-0.03320900	5.33240400
H	1.45501800	-1.72792700	5.90436300
N	0.84854700	0.61486800	-2.16139100
N	1.40614000	-2.03778700	-1.07699700

N	-1.41075100	-1.80408900	-0.28473400
N	-1.03941100	1.96162100	0.97586300
N	1.88685000	-0.56969300	1.40363000
O	-2.28112200	3.76371300	1.96033800
Si	0.33700000	1.91112400	-3.22124300
Si	-2.86950700	-2.24671000	0.57224200
Si	2.57186000	0.29499600	2.76433200
U	0.05048300	0.05576100	0.00445700

$^{IV}TS_{I\text{-}II}$ (triplet)

G= -2177.134730 au

U	0.15917500	0.14617900	-0.21460100
Si	1.26429900	-1.57101600	-3.24889100
Si	-0.06281400	3.77405200	0.89050300
Si	-0.52958800	-2.18748900	2.63579600
N	-0.04378300	-0.76974800	-2.31075400
N	-2.25619200	0.13171100	-0.98237700
N	-0.70369000	2.24705400	0.16348600
N	-0.98572900	-1.30801200	1.12810600
N	1.77060400	0.43874600	0.75289200
C	2.79103100	-0.97663700	-5.68342500
C	1.83446400	1.03191700	-4.48632600
C	1.65723500	-0.46036400	-4.78353000
C	-0.05082700	-3.18943200	-5.33875500
C	-1.26885800	-0.64816400	-3.09660800
C	0.65642200	-3.26837300	-3.97661600
C	-2.24024700	0.32352200	-2.44079300
C	2.93547800	-3.32727700	-1.60001600
C	-1.02767600	5.78389400	-1.05491500
C	1.13409300	4.62364200	-1.60900300
C	4.10045100	-1.32576700	-2.53873600
C	2.75248700	-1.86725600	-2.03967900
C	-0.19821400	-4.09504700	-3.00521600
C	-2.77814400	-1.19073400	-0.58965400
C	0.23034100	5.12102900	-0.47671000
C	-2.92743600	1.24829700	-0.29326900
C	-2.06451100	2.49749600	-0.34410100
C	-2.41185200	-1.51947400	0.85092900
C	-1.39961300	4.54884500	2.06613500
C	-0.86591600	5.78539600	2.80489800
C	2.48001500	4.74529100	1.92202400
C	-0.39066700	-4.61845900	1.04475800
C	1.60446400	3.48808600	1.80767100
C	-2.20242100	-4.64578500	2.77278200
C	-0.80694400	-4.10065700	2.42769300
C	2.29383200	-2.62493700	2.15266200
C	-2.06915900	3.58220300	3.04864800
C	1.43275500	2.82395400	3.18019200
C	1.31764600	-1.85836100	3.05302500
C	-1.71439800	-1.55951800	4.02962200
C	-1.62509500	-0.03953700	4.19999000
C	-1.62476200	-2.25976300	5.39411300
C	1.67612400	-2.10707000	4.52460300

H	2.76975000	-0.47591800	-6.66034300
H	1.88157700	1.61215400	-5.41768200
H	2.73112000	-2.05455600	-5.87006800
H	3.77272600	-0.77614600	-5.24480500
H	0.72974600	-0.55599300	-5.36992200
H	0.56622100	-2.72278000	-6.11135600
H	1.00995300	1.42954500	-3.88652200
H	2.76011600	1.23393800	-3.93798300
H	-0.30751300	-4.19651000	-5.69409400
H	-1.09118400	-0.27316400	-4.11737000
H	-1.76103100	-1.62484200	-3.22864400
H	1.59247300	-3.82586100	-4.13939500
H	-0.98910500	-2.62567800	-5.28255800
H	-3.25523300	0.22058700	-2.85809600
H	3.17053900	-3.97889200	-2.44906100
H	-0.75218800	6.59953700	-1.73736700
H	1.33420800	5.42232900	-2.33634500
H	4.46854100	-1.90676300	-3.39131200
H	-1.90611100	1.34681900	-2.63437200
H	-1.63341900	5.08004400	-1.63667700
H	2.05148600	-3.73538100	-1.10441000
H	0.66643000	3.79589200	-2.15490400
H	-2.31139800	-1.93181800	-1.24250800
H	-3.86923700	-1.23367000	-0.74271600
H	-0.38224700	-5.09961500	-3.40891600
H	-1.67293200	6.21491700	-0.28264900
H	4.05466200	-0.27709400	-2.84208300
H	3.76997500	-3.40827500	-0.89228500
H	2.10068400	4.26493300	-1.24394600
H	-1.17930300	-3.63349500	-2.84491800
H	4.85642100	-1.40428400	-1.74738500
H	-3.92323300	1.42501200	-0.73281200
H	0.26318800	-4.21757200	-2.02337800
H	-2.04557600	2.89366600	-1.37235300
H	0.78073800	5.90909700	0.05997700
H	2.50910500	-1.29516100	-1.12854300
H	-2.72085600	-2.56455900	1.00500500
H	-3.06594000	0.96360300	0.75267600
H	-0.41898900	6.51972800	2.12598000
H	-3.04837900	-0.93122800	1.53346200
H	2.74979100	5.15736100	0.94462000
H	-1.02417500	-4.19440600	0.25775700
H	-2.18742600	4.91589400	1.39147600
H	-2.60077000	3.26139500	0.23557900
H	2.15020700	2.77090900	1.18712800
H	-2.95155800	-4.35253200	2.02798400
H	-0.48691600	-5.71139400	0.98898800
H	3.41779700	4.50443400	2.44011300
H	-1.67429400	6.29340600	3.34806800
H	2.10102700	-2.45735500	1.08934100
H	0.64397800	-4.36752200	0.79794600
H	-0.10510300	5.51558300	3.54495600
H	1.99583400	5.54390100	2.49424900

H	-2.56966300	-4.31333800	3.74708700
H	-2.50704300	2.71633400	2.54234900
H	-2.19147600	-5.74414800	2.78611500
H	-1.72496600	0.48398400	3.24415200
H	3.32557300	-2.30337700	2.34473000
H	-2.87507700	4.08357900	3.60222200
H	2.25104300	-3.70492100	2.33792700
H	-2.71402100	-1.78663900	3.62694700
H	0.84112600	1.90651300	3.11770500
H	1.46691700	-0.78994200	2.85062800
H	-0.10784600	-4.52867700	3.16472900
H	-1.36146200	3.19589000	3.78801800
H	2.41056300	2.55408300	3.60003500
H	0.94768300	3.49309500	3.89966200
H	-2.40547600	0.33680200	4.87510700
H	-0.65983400	0.25553500	4.62853300
H	-1.58872700	-3.35087800	5.31816800
H	1.44748600	-3.13080700	4.84631100
H	-2.49369000	-2.00457300	6.01553000
H	2.75276300	-1.95761200	4.68092200
H	1.15560100	-1.42255600	5.19921800
H	-0.73687200	-1.94780800	5.95065500
C	2.12738000	1.32697100	-0.97026400
O	3.17819600	1.79240800	-1.09716700

CO

G= -113.272375 au

C	0.00000000	0.00000000	-0.64972200
O	0.00000000	0.00000000	0.48729100

References

1. D. M. King, F. Tuna, E. J. L. McInnes, J. McMaster, W. Lewis, A. J. Blake, S. T. Liddle, *Science* **2012**, *337*, 717.
2. D. M. King, F. Tuna, E. J. L. McInnes, J. McMaster, W. Lewis, A. J. Blake, S. T. Liddle, *Nat. Chem.* **2013**, *5*, 482.
3. D. E. Bergbreiter, J. M. Killough, *J. Am. Chem. Soc.* **1978**, *100*, 2126.
4. C. Fonseca Guerra, J. G. Snijders, G. te Velde E. J. Baerends, *Theor. Chem. Acc.* **1998**, *99*, 391.

5. G. te Velde, F. M. Bickelhaupt, S. J. A. van Gisbergen, C. Fonseca Guerra, E. J. Baerends, J. G. Snijders T. Ziegler, *J. Comput. Chem.* **2001**, *22*, 931.
6. S. H. Vosko, L. Wilk, M. Nusair, *Can. J. Phys.* **1980**, *58*, 1200.
7. A. D. Becke, *Phys. Rev. A.* **1988**, *38*, 3098.
8. J. P. Perdew, *Phys. Rev. B.* **1986**, *33*, 8822.
9. A. D. Becke, *J. Chem. Phys*. **1993**, *98*, 5648.
10. J. P. Perdew, Y. Wang, *Phys. Rev. B* **1992**, *45*, 13244.
11. W. Kuchle, M. Dolg, H. Stoll, H. Preuss, *J. Chem. Phys*. **1994**, *100*, 7535.
12. X. Y. Cao, M. Dolg, H. Stoll, *J. Chem. Phys*. **2003**, *118*, 487.
13. X. Y. Cao, M. Dolg, *J. Molec. Struc. (Theochem)* **2004**, *673*, 203.
14. A. Bergner, M. Dolg, W. Kuechle, H. Stoll, H. Preuss, *Mol. Phys.* **1993**, *80*, 1431.
15. A. W. Ehlers, M. Böhme, S. Dapprich, A. Gobbi, A. Höllwarth, V. Jonas, K. F. Köhler, R. Stegmann, A. Veldkamp, G. Frenking, *Chem. Phys. Lett.* **1993**, *208*, 111.
16. R. Ditchfield, W. J. Hehre, J. A. Pople, *J. Chem. Phys.* **1971**, *54*, 724.
17. W. J. Hehre, R. Ditchfield, J. A. Pople, *J. Chem. Phys.* **1972**, *56*, 2257.
18. P. C. Hariharan, J. A. Pople, *Theor. Chim. Acta.* **1973**, *28*, 213.
19. C. Gonzalez, H. B. Schlegel, *J. Chem. Phys.* **1989**, *90*, 2154.
20. C. Gonzalez, H. B. Schlegel, *J. Phys. Chem.* **1990**, *94*, 5523.
21. Gaussian 09, Revision D.01, M. J. Frisch, G. W. Trucks, H. B. Schlegel, G. E. Scuseria, M. A. Robb, J. R. Cheeseman, G. Scalmani, V. Barone, B. Mennucci, G. A. Petersson, H. Nakatsuji, M. Caricato, X. Li, H. P. Hratchian, A. F. Izmaylov, J. Bloino, G. Zheng, J. L. Sonnenberg, M. Hada, M. Ehara, K. Toyota, R. Fukuda, J. Hasegawa, M. Ishida, T. Nakajima, Y. Honda, O. Kitao, H. Nakai, T. Vreven, J. A. Montgomery, Jr., J. E. Peralta, F. Ogliaro, M. Bearpark, J. J. Heyd, E. Brothers, K. N. Kudin, V. N. Staroverov, R. Kobayashi, J. Normand, K. Raghavachari, A. Rendell, J. C. Burant, S. S. Iyengar, J. Tomasi, M. Cossi, N. Rega, J. M. Millam, M. Klene, J. E. Knox, J. B. Cross, V. Bakken, C. Adamo, J. Jaramillo, R. Gomperts, R. E. Stratmann, O. Yazyev, A. J. Austin, R. Cammi, C. Pomelli, J. W. Ochterski, R. L. Martin, K.

Morokuma, V. G. Zakrzewski, G. A. Voth, P. Salvador, J. J. Dannenberg, S. Dapprich, A. D. Daniels, Ö. Farkas, J. B. Foresman, J. V. Ortiz, J. Cioslowski, and D. J. Fox, Gaussian, Inc., Wallingford CT, 2009.
